# Detection of Volatile Metabolites Derived from Garlic (*Allium sativum*) in Human Urine

**DOI:** 10.3390/metabo6040043

**Published:** 2016-12-01

**Authors:** Laura Scheffler, Yvonne Sauermann, Anja Heinlein, Constanze Sharapa, Andrea Buettner

**Affiliations:** 1 Department of Chemistry and Pharmacy, Emil Fischer Center, Friedrich-Alexander-Universität Erlangen-Nürnberg (FAU), Henkestr. 9, 91054 Erlangen, Germany; laura.scheffler@fau.de (L.S.); Yvonne.sauermann@fau.de (Y.S.); anja.heinlein@fau.de (A.H.); 2 Fraunhofer Institute for Process Engineering and Packaging (IVV), Giggenhauser Str. 35, 85354 Freising, Germany; constanze.sharapa@fau.de

**Keywords:** garlic, human urine, gas-chromatography mass-spectrometry/olfactometry, allyl methyl sulfide, allyl methyl sulfoxide, allyl methyl sulfone

## Abstract

The metabolism and excretion of flavor constituents of garlic, a common plant used in flavoring foods and attributed with several health benefits, in humans is not fully understood. Likewise, the physiologically active principles of garlic have not been fully clarified to date. It is possible that not only the parent compounds present in garlic but also its metabolites are responsible for the specific physiological properties of garlic, including its influence on the characteristic body odor signature of humans after garlic consumption. Accordingly, the aim of this study was to investigate potential garlic-derived metabolites in human urine. To this aim, 14 sets of urine samples were obtained from 12 volunteers, whereby each set comprised one sample that was collected prior to consumption of food-relevant concentrations of garlic, followed by five to eight subsequent samples after garlic consumption that covered a time interval of up to 26 h. The samples were analyzed chemo-analytically using gas chromatography-mass spectrometry/olfactometry (GC-MS/O), as well as sensorially by a trained human panel. The analyses revealed three different garlic-derived metabolites in urine, namely allyl methyl sulfide (AMS), allyl methyl sulfoxide (AMSO) and allyl methyl sulfone (AMSO_2_), confirming our previous findings on human milk metabolite composition. The excretion rates of these metabolites into urine were strongly time-dependent with distinct inter-individual differences. These findings indicate that the volatile odorant fraction of garlic is heavily biotransformed in humans, opening up a window into substance circulation within the human body with potential wider ramifications in view of physiological effects of this aromatic plant that is appreciated by humans in their daily diet.

## 1. Introduction

Garlic (*Allium sativum*) is a well-known aromatic plant used in everyday cuisine. According to the Agricultural Marketing Resource Center the annual per capita retail consumption of garlic was 1.2 pounds (~0.54 kg) in 1991 in the USA, whereas in 2001 the retail consumption raised to 2 pounds (~0.9 kg), i.e., almost doubling over a 10-year period [1]. This increase might relate to increased awareness of the beneficial health properties of garlic. To the best of our knowledge, research on garlic flavor started as early as the 1930s [2]; since then garlic has become one of the most researched plant food materials. In the course of these research activities, several health effects have been associated with garlic consumption, e.g., reduction of blood pressure, improvement of cholesterol levels, and decrease in blood triacylglyceride levels. Since high blood pressure, a high total cholesterol (TC) level and a low high density lipoprotein (HDL)/low density lipoprotein (LDL)-ratio are the main risk factors for cardiovascular diseases (CVD), garlic is believed to have a protective effect against such impacting factors [3,4,5,6,7]. 

Most of the garlic constituents responsible for these effects are not yet known, although the active compounds saponins and flavonoids of the garlic bulb have been proposed [8,9,10]. Another compound that has been reported to be responsible for the effects of garlic is allicin and its (unspecified) degradation products [11]. Allicin itself is not present in the intact garlic bulb but is generated by the enzyme alliinase in the course of cell disruption, e.g., by chewing or cutting; the precursor substance is the non-proteinogenic amino acid alliin (S-allyl-L-cysteine sulfoxide) [12]. Allicin is then further degraded, thereby generating a multitude of compounds including disulfides, trisulfides, dithiins and ajoenes [13,14,15,16]. These compounds constitute the typical aroma of garlic but may also contribute to its beneficial health effects. 

Recent research has demonstrated that volatiles may cause physiological effects. Aroma compounds have been shown to modulate different types of receptors beyond the olfactory system, such as the γ-aminobutanoic acid (GABA_A_)-receptor [17,18] and the vanilloid transient receptor potential cation channel (TRPV1)-receptor [19]. Potential physiological effects in relation to food or beverages are often investigated on substances that occur naturally in the product, but potential metabolism within the body is rarely considered. In view of this, recent research has shown that volatile substances can undergo major transformation steps within the body or can be released from non-volatile precursors, e.g., in the human gastrointestinal tract [20] or during absorption processes [21], leading to compounds that were not present in the original product [22,23,24,25]. 

Regarding garlic aroma, the metabolism of its constituents in the human body is not fully understood. This issue has previously been addressed in animal or tissue model studies, leading to the discovery of allyl mercaptan, allyl methyl sulfide (AMS), diallyl disulfide (DADS) and diallyl sulfone (DASO_2_) as possible metabolites [13,26,27,28,29,30,31]. In a recent study, we were able to identify three garlic metabolites in human milk, namely AMS, allyl methyl sulfoxide (AMSO) and allyl methyl sulfone (AMSO_2_).

To complement these findings, the present study aimed to investigate the volatile garlic-derived metabolites in human urine over time after consumption of garlic at dietary relevant concentration.

## 2. Results

### 2.1. Sensory Analysis

Changes in the odor of bodily fluids may indicate the presence of volatile constituents or metabolites of food components [22,23,25]. Pre-trials indicated that slight odor changes, relating to the attribute “garlic-/cabbage-like”, were observable in human urine after garlic consumption; accordingly, this attribute was rated by the panelists in the different urine samples on a scale from 0 (no perception) to 3 (strong perception). In the case of the high dosage test (Urine *a*), where the test person consumed about 30 g of raw garlic, a “garlic-/cabbage-like” odor was rated only as weak, with an average value of 0.6 in the sample that was obtained about 2 h after garlic consumption, subsequently decreasing to 0.5 and 0.4 in the third and fourth urine sample of the set. The first urine sample of this set, which was obtained prior to garlic consumption, was also rated as having a very slight “garlic-/cabbage-like” odor (0.3). However, these results are only from a single test subject. Sensory analysis of the other urine sets where each test person consumed about 3 g of raw garlic indicated that a slight “garlic-/cabbage-like” odor was perceivable during the first hours after garlic consumption, sometimes being perceivable up to 24 h later. The intensities of this odor ranged from 0 to 0.7, corresponding to a very weak smell. The perceived intensities of a “garlic-/cabbage-like” odor in urine at different time intervals before and after ingestion of raw garlic are shown in supplementary Figure S1.

### 2.2. High Resolution Gas Chromatography-Olfactometry (HRGC-O) and Comparative Aroma Extract Dilution Analysis (cAEDA) of Urine before and after Garlic Consumption

For identification of potentially odor active compounds in urine deriving from garlic consumption, the solvent extracts of the urine samples of Urine *a* were analyzed by HRGC-O using a capillary DB-5 as well as a capillary DB-FFAP. For all odor active substances, that could be perceived at the sniffing port, the retention indices (RI) according to Van den Dool and Kratz [32] were calculated. Using this approach two substances with a garlic-like odor were detected via HRGC-O. One compound had RIs of 715 on the DB-5 capillary and <1000 on the DB-FFAP capillary, whereas the other had RIs of 973 and 1362 on the DB-5 and DB-FFAP capillaries, respectively. The first compound was identified as AMS and the second as dimethyl trisulfide (DMTS), as based on a comparison of odor qualities and RIs of pure reference compounds. No additional substances that could directly be related to garlic consumption were identified. All remaining odorous molecules detected were also present in the control samples, indicating that these were common urine odor constituents (data not shown).

The cAEDA confirmed the presence of AMS and DMTS in the urine sample set *b* to *f*, as summarized in Table 1. No additional odorous substances that could be attributed to the garlic intervention were detected, with all remaining odorous molecules also being present in the control samples (data not shown). These substances were different lactones such as γ-nonalactone or δ-decalactone, and several guaiacol-derivatives, as have been previously reported to be present in urine [33,34,35]. Accordingly, these compounds are not described in further detail here.

AMS was only detected in samples after garlic consumption, but DMTS was also detected in some samples prior to garlic consumption, and with comparable flavor dilution (FD) factors. Furthermore, DMTS was not always detected over the entire sampling period, in some cases disappearing and reappearing at later times. Moreover, in some cases the urine sample collected 24 h after garlic ingestion had a higher FD factor for DMTS.

Based on the finding that only AMS and DMTS were detectable as additional odorous compounds after garlic consumption and that the appearance of DMTS was not only related to garlic consumption, a shortened cAEDA was performed on the extracts of urine samples of the sets *g* to *n*, whereby only the first five minutes of each GC-run were analyzed. The FD factors obtained for AMS for the sample sets Urine *g* to Urine *n* are provided in the supplementary Table S1. 

### 2.3. Identification of Garlic-Derived Metabolites in Urine 

#### 2.3.1. Identification Using HRGC-MS

Subsequently, targeted analyses via HRGC-MS were performed to further confirm the olfactometric identification of AMS and DMTS, and to screen for potential further garlic-derived metabolites that are odorless or less odor active and therefore not detectable by HRGC-O. To this aim, the extracts of the sample series of Urine *a* and *b* were analyzed both on capillary DB-5 and DB-FFAP. The obtained chromatograms were compared and matched with their respective control samples, so that additional peaks in the urine extracts resulting from garlic intervention could be elaborated. Additionally, a targeted search was carried out for those substances that have either been reported to be present in garlic itself or have been previously proposed as metabolites [22,29,36,37,38,39,40]. This target substance selection is shown in Table 2, together with the RIs and *m*/*z* ratios used for data extraction.

**Figure 1:**
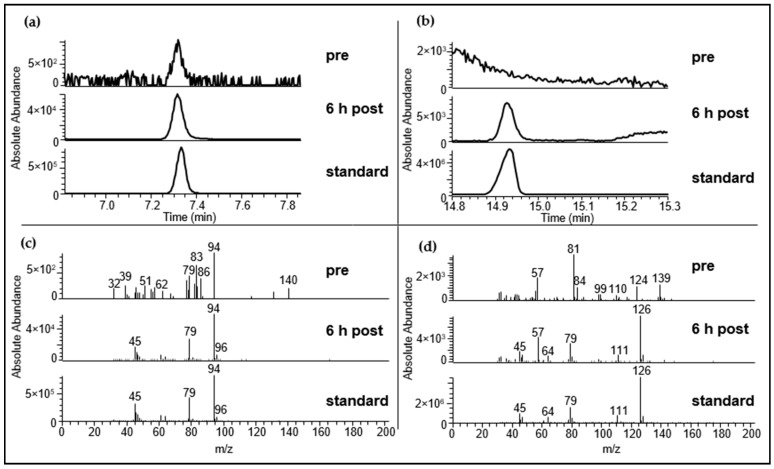
Identification of dimethyl disulfide (DMDS) and dimethyl trisulfide (DMTS) in urine samples was performed via HRGC-MS. (**a**): Extracted ion chromatogram (*m*/*z* 94) of DMDS in an urine sample that was collected prior to garlic consumption (*pre*) and in an urine sample that was obtained after garlic consumption (*6 h post*); (**b**): Extracted ion chromatogramm (*m*/*z* 126) of DMTS in an urine sample that was collected prior to garlic consumption (*pre*) and in an urine sample that was obtained after garlic consumption (*6 h post*); (**c**): Mass spectra of DMDS in urine sample that was collected prior garlic consumption (*pre*) and after garlic consumption (*6 h post*) and a DMDS-standard (*standard*); (**d**): mass spectra of DMTS in urine sample that was collected prior garlic consumption (*pre*) and after garlic consumption (*6 h post*) and a DMTS-standard (*standard*). Exemplarily urine sample *b* is displayed.

The presence of DMTS was confirmed in the majority of samples (84 of 105 urine samples; 80.0%), although this substance was often present in only trace amounts. Additionally, DMDS was identified in a series of samples, as shown in Figure 1 for two samples of urine set *b*. In this case, DMDS was detected in almost all samples collected after garlic consumption, as well as in trace amounts in the sample obtained prior to garlic consumption (89 of 105 urine samples; 84.8%). The detected amounts of DMDS and DMTS in all urine samples and the corresponding sampling times of the respective samples are given in the supplementary Table S2.

By comparison, AMS could not be determined with sufficient resolution by one-dimensional GC-MS analysis due to co-elution of substances; accordingly, identification of this compound was based on two-dimensional GC-MS analyses, as described in Section 2.3.2. 

**Figure 2:**
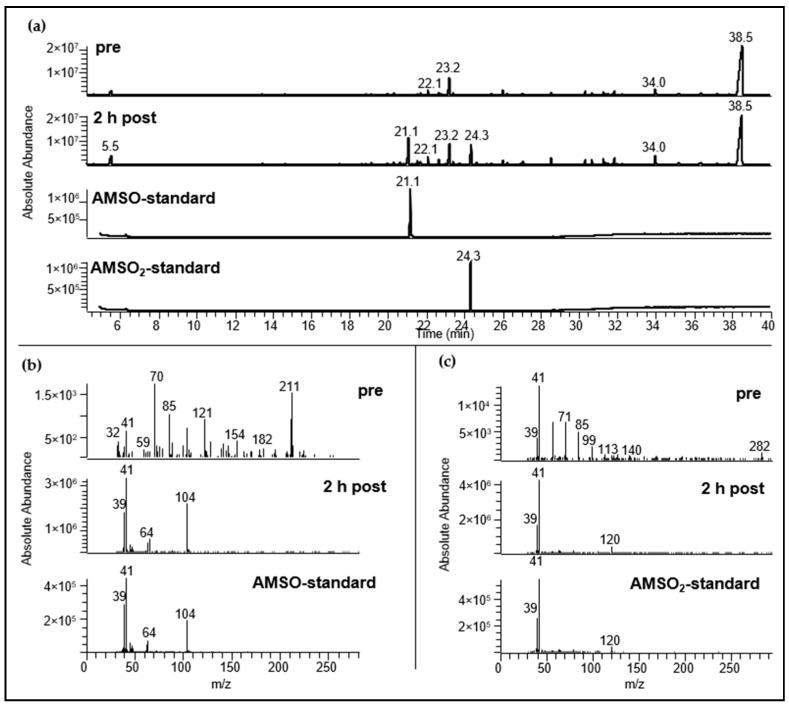
Identification of garlic-derived compounds in urine samples was performed via HRGC-MS. (**a**): Total ion chromatogram (TIC) of urine samples before (*pre*) and 2.2 h after garlic consumption (*2 h post*), AMSO reference standard (*AMSO standard*) and AMSO_2_ reference standard (*AMSO*_2_*-standard*). The volunteer consumed about 30 g of raw garlic. Temperature program: 40 °C, held for 7 min, raised with 8 °C/min to 240 °C and held for 8 min; (**b**): mass spectra of AMSO in urine sample before garlic consumption (*pre*), after garlic consumption (*2 h post*) and AMSO reference standard (*AMSO-standard*); (**c**): mass spectra of AMSO_2_ in urine sample before garlic consumption (*pre*), after garlic consumption (*2 h post*) and AMSO_2_-reference standard (*AMSO_2_-standard*). The mass spectra are shown at the time when the standard compound eluted.

Two additional substances that were not perceivable via GC-O were detected using HRGC-MS: Allyl methyl sulfoxide (AMSO) and allyl methyl sulfone (AMSO_2_). In the high dosage experiment (Urine *a*) both compounds were detectable as two additional peaks in the total ion chromatogram (TIC). Figure 2 displays the TIC of the urine sample prior to garlic consumption (Figure 2a-*pre*) together with the TIC of the urine sample that was collected 2.1 h after garlic consumption (Figure 2a-*2 h post*). In addition, the chromatograms of the reference substances AMSO and AMSO_2_ together with their respective mass spectra (Figure 2b,c) are displayed, confirming unambiguous identification of either compound. The presence of AMSO and AMSO_2_ in the sample collected prior to garlic consumption could thereby be excluded (cf. Figure 2a *pre*, Figure 2b *pre*, Figure 2c *pre*).

#### 2.3.2. Identification Using HRGC-GC-MS

**Figure 3:**
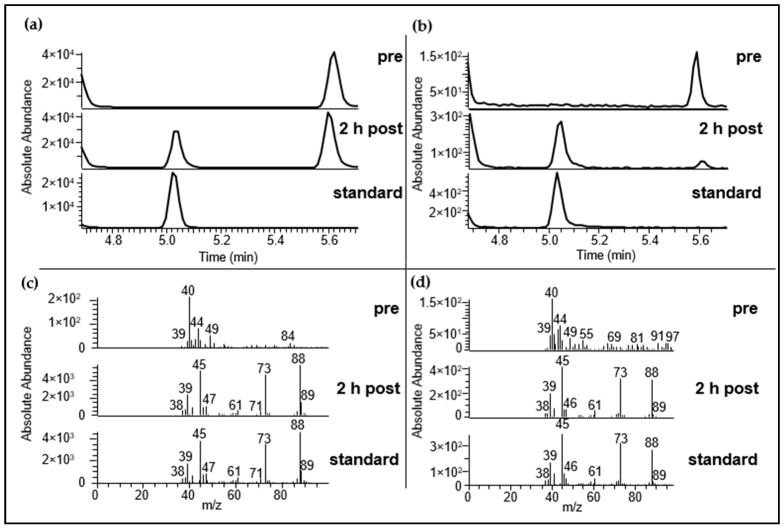
Identification of AMS in urine was performed via HRGC-GC-MS using capillary DB-5 as well as capillary DB-FFAP. 4 µL were applied for analyses. (**a**): TIC of urine samples before (*pre*) and 2.2 h after garlic consumption (*2 h post*) as well as an AMS-Standard (conc. 5 µg/mL) (*standard*). Test person consumed about 30 g of raw garlic; (**b**): Extracted ion chromatogram (*m*/*z* 73 + 88) of urine sample before (*pre*) and 2.1 h after garlic consumption (*2 h post*) as well as an AMS-Standard (conc. 0.25 µg/mL) (*standard*). Test person consumed about 3 g of raw garlic. Exemplarily urine sample *b* is displayed; (**c**) Respective mass spectra of AMS for the samples shown in (**a**); (**d**) Respective mass spectra of AMS for the samples shown in (**b**). The mass spectra are shown at the time when the standard compound eluted.

As previously discussed, an unambiguous detection of AMS by means of HRGC-MS was not achievable due to interference with co-eluting substances. Accordingly, a two dimensional HRGC-GC-MS system was applied for increased selectivity and sensitivity in the detection of the target compound. Using this approach, AMS was successfully confirmed in the urine samples that were obtained both after consumption of 30 g as well as 3 g of raw garlic. Thereby, no extraction of specific *m*/*z* was necessary for the high dosage experiment, whereas for the lower dosage experiments *m*/*z* 73 + 88 were extracted from the TIC for targeted detection of AMS. Moreover, the presence of AMS in urine samples collected prior to garlic consumption could be excluded (cf. Figure 3).

In addition to AMS, the urine samples were screened for compounds that have previously been identified in garlic or proposed as potential garlic metabolites. These substances were diallyl sulfoxide (DASO), DASO_2_, DADS, allyl methyl disulfide (AMDS), dimethyl disulfide (DMDS), DMTS, diallyl trisulfide (DATS), diallyl sulfide (DAS), 2-vinyl-4H-1,3-dithiin and 3-vinyl-4H-1,2-dithiin (see Table 2). Urine sample U *a 4 h post* was used to search for the presence of these compounds, since the highest concentrations of excreted substances and potential metabolites could be expected in this sample. A targeted search was conducted in comparison to the respective reference standards by parallel analyses applying the same analytical conditions. As compiled in Table 2, most of the substances were not detectable in the urine sample whereas the presence of AMDS, DMDS and DMTS could be confirmed. DMTS was also detected via HRGC-MS, as discussed above. In a second experiment, it was screened for the presence of AMDS and DMDS in urine sample *f* obtained 2 h after garlic consumption; this choice was based on the consideration that the excretion of the garlic metabolites AMS, AMSO and AMSO_2_ reached a maximum at this time for this sample set (see Section 2.5.1). Neither AMDS nor DMDS were detectable in U *f 2 h post*, whereas DMDS was successfully detected in urine set *b* over the course of the identification experiments, as described in Section 2.3.1.

### 2.4. Difference between Concentrations of the Garlic-Derived Metabolites Depending on Calculation Method

**Figure 4:**
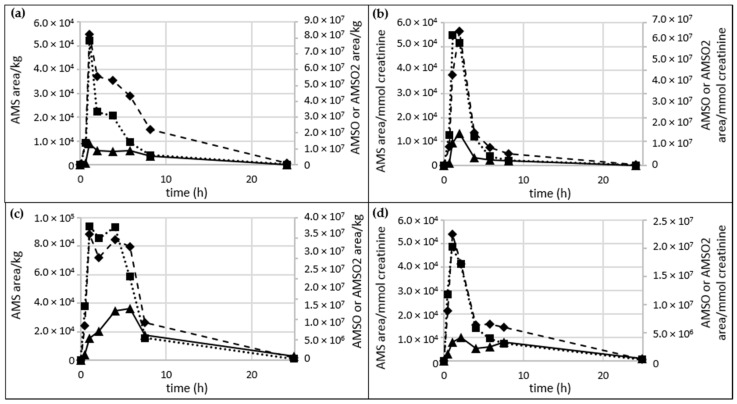
Difference between calculation methods of the concentrations of the garlic-derived metabolites. (**a**) and (**c**): concentrations of garlic-derived metabolites in Urine *c* and Urine *d*, calculated as area/kg; (**b**) and (**d**): concentrations of garlic-derived metabolites in Urine *c* and Urine *d*, calculated as area/mmol creatinine. Areas are based on the *m*/*z*-ratios 73 + 88, 104 and 120 for AMS, AMSO and AMSO_2_, respectively. AMS (◆), AMSO (■), AMSO_2_ (▲).

The concentrations of constituents in urine are highly dependent on the water intake of the volunteer. This also applies to garlic-derived metabolites. Accordingly, the concentrations of AMS, AMSO and AMSO_2_ were calculated in two different ways, as area/kg and normalized to the creatinine content of the urine sample. Two urine sets of test persons *c* and *d* are shown in Figure 4. 

A distinct difference between the two calculation methods is observable in Figure 4a,c, in which the amount of the garlic-derived metabolites was calculated as area/kg. AMS and AMSO show their maxima about 1 h after garlic consumption and a second distinct increase 2 to 4 h after garlic consumption. Likewise, a maximum at about 1 h after garlic intervention can be observed for AMSO_2_ for sample set *c*. For sample set *d* there was a continuous increase until 6 h after garlic consumption; AMSO_2_ decreased only in the last two samples, collected about 8 and 24 h after garlic consumption. The creatinine-normalized values showed distinct maxima in AMS, AMSO and AMSO_2_ 1 to 2 h after garlic consumption. A second increase of the metabolites was observed for Figure 4d, but this was not as distinct as in Figure 4c. No second increase was observable in Figure 4b.

A table listing the creatinine content, amount of urine, areas of the respective metabolites and calculated amounts of metabolites (in area/kg as well as area/mmol creatinine) for all urine samples is provided in the supplementary Table S2 (DMTS and DMDS) and Table S3 (AMS, AMSO and AMSO_2_).

### 2.5. Time Dependency of Appearance of Specific Garlic-Derived Metabolites in Urine after Consumption of Garlic 

#### 2.5.1. AMS, AMSO and AMSO_2_

In order to monitor the temporal profiles of the identified garlic-derived metabolites in human urine in more detail, a relative semi-quantitative estimation was performed. For this aim the area/mmol creatinine ratios were calculated and plotted versus the sampling time of the respective urine sample. 

**Figure 5:**
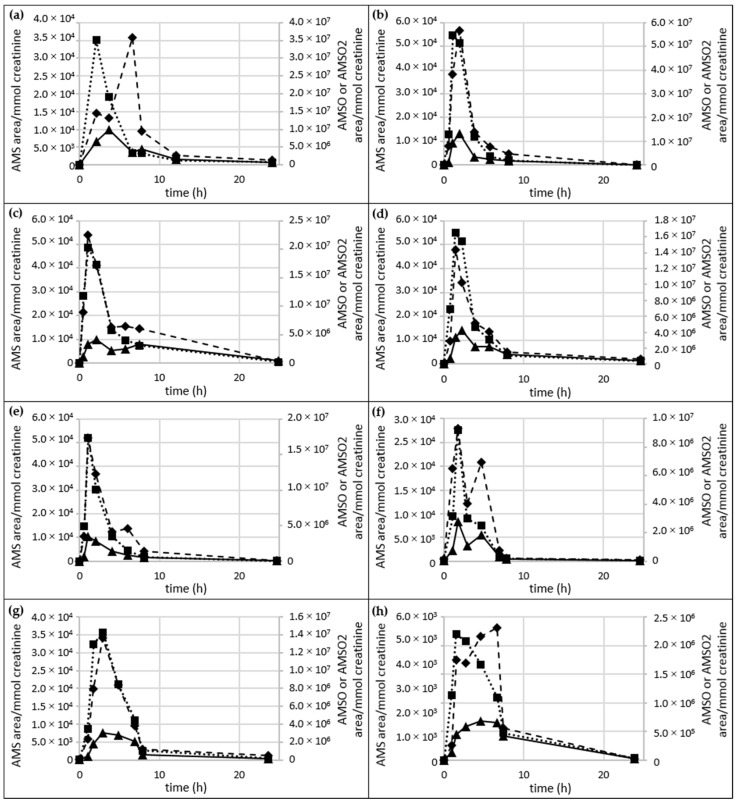

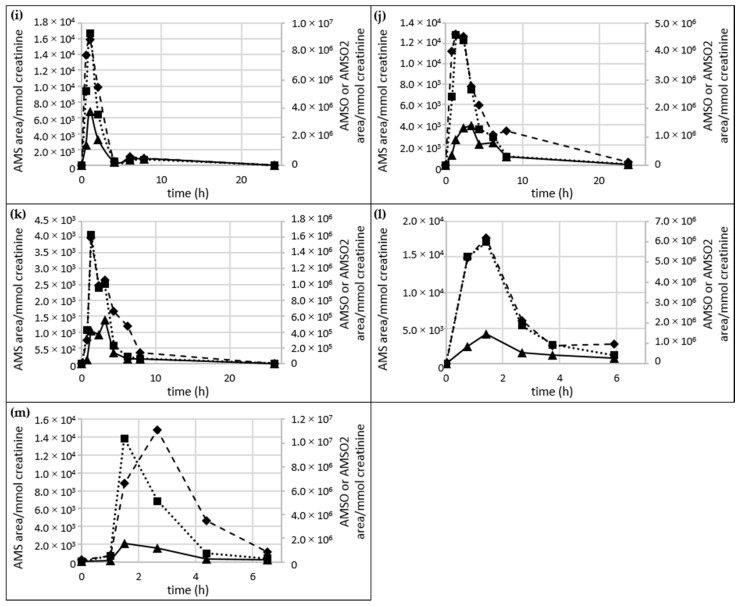
Time-Resolved metabolite profiles of AMS, AMSO and AMSO_2_. 12 urine sample sets; only samples *b* and *e* were provided by the same test person, albeit at different days. AMS (◆), AMSO (■), AMSO_2_ (▲), time 0 h represents the urine sample collected prior to garlic consumption, subsequent time intervals represent urine samples after garlic consumption. Garlic was consumed between 2 to 38 min after the first urine sample was collected. (**a**) set *b*; (**b**) set *c;* (**c**) set *d*; (**d**) set *e*; (**e**) set *f* (**f**) set *g*; (**g**) set *h;* (**h**) set *i*; (**i**) set *j*; (**j**) set *k*; (**k**) set *l*; (**l**) set *m*; (**m**) set *n.*

The amounts of AMS, AMSO and AMSO_2_ in urine sets *b* to *n* are shown in Figure 5. A table with the exact values of the metabolites is provided in the supplementary Table S3. In the case of urine set *a,* the metabolites were calculated as area/kg, since creatinine levels were not measured during this high-dosage test. This set differed from the others (3 g garlic consumption), in so far as it was characterized by a continuous increase over the entire sampling period (5.8 h); only AMSO decreased slightly at the last point of determination. A figure displaying the metabolite profile of the high dosage test is provided in supplementary Figure S2.

- *Urine series b–l*:
  For most of the urine sets (Urine *f, g, h, j, l*) the maximum concentration of all three garlic-derived metabolites, AMS, AMSO and AMSO_2_, approximately coincided, namely at about 1 h after garlic consumption. This maximum was observed at about 2.4 h after garlic consumption in sample set *h* only. The maxima in AMS and AMSO for sets *b, d, e, i* and *k* were reached before AMSO_2_. AMS and AMSO showed the highest values at about 1 to 1.5 h after garlic consumption (at 2 h for sample set *b*), whereas AMSO_2_ was observed to peak at about 2 to 3 h (4.5 h for sample set *i*). AMSO reached its maximum before AMS and AMSO_2_ only in sample set *c*. Whereas AMSO was excreted mostly at about 0.9 h after garlic consumption, AMS and AMSO_2_ reached their highest values at about 1.8 h after garlic consumption. Apart from the first maximum, a second increase in the metabolites was observed in several cases, which differed in time as well as intensity between the sample sets, indicating large inter-individual differences in the metabolism and excretion rate of garlic constituents. In most cases the second increase was smaller than the first; this was the case for AMS in sets *d, f, g, i, j, k* and *l*, for AMSO in sets *b, j* and *l*, and for AMSO_2_ in sets *d, e, g, j, k* and *l*. On the other hand, the second increase was more distinct than the first for AMS in two sample sets (*b, i*) and for AMSO_2_ in set *l*. No second increase was observed for all three garlic-derived metabolites in set *c,* for AMS in sets *e* and *h*, for AMSO in sets *d, e, f, g, h* and *k*, and for AMSO_2_ in sets *b, f* and *h*. In some cases the formation of a “shoulder” was observed for AMS (sets *e* and *h*), AMSO (sets *g, h* and *k*), and AMSO_2_ (sets *b* and *h*) at time intervals of 3 h (set *l*) to 7.8 h (set *k*) after garlic consumption.
- *Urine series m–n*:
  Urine sample sets *m* and *n* were investigated over a period of about 6 h, comprising six different samples. Only one maximum was measured for each metabolite during the sampling period. For sample set *m* all three metabolites reached their maximum at about 1.5 h after garlic ingestion, whereas for sample set *n* maximum concentrations of both AMSO and AMSO_2_ were excreted at about 1.5 h but AMS reached its maximum at about 2.5 h after garlic ingestion.

#### 2.5.2. DMTS and DMDS 

**Figure 6:**
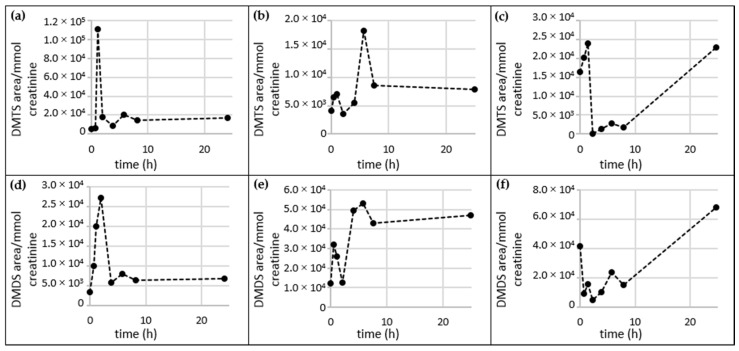
Time-resolved metabolite profiles of DMTS and DMDS. Urine sample sets from three volunteers (set *c*, *d* and *e*). (**a**)–(**c**): DMTS; (**d**)–(**f**): DMDS, time 0 h represents the urine sample collected prior to garlic consumption, subsequent time points represent urine samples after garlic consumption. Garlic was consumed 10 to 22 min after the first urine sample was collected.

The excretion profiles of DMTS and DMDS were monitored in order to determine whether their excretion directly related to garlic consumption. Example profiles are shown in Figure 6. Further profiles are provided in the supplementary Figure S3 (DMTS) and Figure S4 (DMDS).

Large inter-individual differences were observed in the excretion profiles of both DMTS and DMS. A distinct maximum of these two compounds appeared about 1 and 1.8 h after garlic consumption in sample set *c*. For sample set *d* both compounds were mostly excreted at about 5.7 h after garlic consumption. In contrast to Urine *c* and *d*, an increase in DMTS and DMDS was observed in sample set *e* that did not decrease over the period of observation. DMTS and DMDS were also measurable in most of the urine samples collected prior to garlic consumption (see supplementary Table S2).

## 3. Discussion

### 3.1. Sensory Analysis

Sensory analyses revealed that the smell of urine changes slightly after garlic consumption, exhibiting a weak garlic-like and cabbage-like odor. This change was distinct in urine samples collected after consumption of 30 g raw garlic, but in other cases (3 g garlic consumption) the change was not as clear, and a faint garlic-like impression was perceivable even in several samples collected prior to garlic consumption. This indicates that the odor is not only related to the consumption of garlic but also other substances that are excreted into urine.

The lack of a pronounced garlic odor in urine might be due to its very low perceived intensity (always rated below 1), making it difficult to detect unambiguously. Accordingly, this odor impression was potentially covered by other smells of the urine. Additionally, some substances exhibiting a garlic-like, sulfury smell have previously been described as odorous constituents of human urine, irrespective of garlic consumption. Wagenstaller and Buettner [33], for example, identified DMTS as a common odorous constituent in several urine samples, and Wahl et al. [43] described several sulfur-containing compounds, such as methyl propyl disulfide, methyl-2-propenyl disulfide, DMDS and DMTS in the urine of healthy subjects without garlic intervention. These findings are confirmed by Mills et al. [44], who additionally identified methanethiol in human urine, and by Smith et al. [45]. DMDS and DMTS specifically exert a cabbage- or garlic-like smell [41,42] and their presence in urine does not necessarily depend on garlic-consumption.

Contrary to our findings in urine, in our previous study we perceived a clear garlic-/cabbage-like odor in human milk after garlic ingestion. This was related to the excretion of AMS, whereas such a clear relationship could not be established for urine. The likely reason is the high inherent smell of urine compared to human milk, which exhibits a very low overall smell intensity. AMSO and AMSO_2_ are odorless and do not contribute to the odor of either urine or milk.

### 3.2. Identification of Garlic-Derived Metabolites in Human Urine

In the present study, the investigation of urinary constituents that are associated with garlic consumption led to the successful identification of three garlic-derived metabolites in human urine, namely AMS, AMSO and AMSO_2_. 

AMS has previously been reported as a compound in human breath and urine after garlic consumption [26,37,38,46], as well as a constituent of garlic itself [41,42,47,48,49]. In contrast, the derivatives AMSO and AMSO_2_ are reported here for the first time as garlic-derived compounds in human urine, demonstrating that constituents of garlic are heavily metabolized prior to their excretion via urine. It is also interesting to note that AMS, AMSO and AMSO_2_ have previously been reported as garlic-derived constituents in human milk by our group [22], indicating that there are similar pathways for the excretion of such substances via different bodily fluids. 

The presence of relevant amounts of AMS, AMSO and AMSO_2_ in urine samples collected prior to garlic consumption could be generally ruled out, with these garlic-metabolites being detectable at only trace amounts in the blank urine samples of sets *g*, *h* and *n*. It is interesting to note that, according to the dietary records, these volunteers consumed olives during the wash-out days (see supplementary Tables S9 and S10). Like garlic, olives are characterized by a relatively high sulfur content; Fleming et al. [50] identified DMS as a major odorous compound in olives, and Collin et al. [51] detected several sulfur-containing compounds in olives. Accordingly, it is possible that olive constituents are metabolized in a similar way as garlic compounds, potentially explaining the presence of trace amounts of sulfury metabolites in the blank urine samples. As reported in our previous study on human milk, volunteer *n* exhibited traces of garlic substances in both milk and urine samples, which is suspected to relate to this subject consuming some tomato sauce of unknown composition in a restaurant on the day prior to sampling (see supplementary Table S16), thus the ingestion of garlic could not be fully excluded. Nevertheless, in relation to the trace amounts that were detected in the blank samples, a major increase of the metabolites in the following samples was observed, confirming a clear correlation between garlic consumption and the excretion of AMS, AMSO and AMSO_2_. 

The present study further focused on those compounds that have been previously reported as being constituents of garlic or potential metabolites. With regards to the compounds that are present in garlic itself (DADS, AMDS, DMDS, DMTS, DATS, DAS, 2-vinyl-4H-1,3-dithiin, 3-vinyl-4H-1,2-dithiin) [39,40,41,42,47,48,49,52,53] it was found that solely DMDS and DMTS were detectable in human urine after garlic consumption, albeit with some cases of detection of these compounds prior to garlic consumption. DMDS and DMTS have been described earlier as compounds that can naturally occur in urine [33,43,44,45]. Regarding the temporal profiles of DMDS and DMTS monitored in the present study, no clear pattern was found. In most sets of urine a maximum in the temporal excretion profiles of both compounds was detected over the sampling period, with the time of the maximum ranging from 1 to 8 h after garlic consumption. In one case DMDS and DMTS reached their maxima at about 24 h after garlic consumption (Urine *e*). Accordingly, other sulfur-containing compounds from other dietary sources are likely to be metabolized and excreted in urine in the form of DMDS and DMTS. It is interesting to note here that DMTS has recently been reported to be present in cranberries [54], indicating that this compound is not restricted to Allium species. Nevertheless, an additional influence of garlic consumption cannot be fully ruled out. 

Other compounds that have been previously detected in human breath after administration of garlic are methyl mercaptan, allyl mercaptan, dimethylsulfide, DADS, AMDS, DMDS, DATS, and DAS [36,37,38,46,55,56,57,58,59,60,61]. However, studies by Taucher et al. [46], Suarez et al. [38] and Buhr et al. [55] indicate that solely AMS and dimethylsulfide are metabolites of garlic constituents whereas the other compounds might originate from garlic residues in the oral cavity. In the present study neither DADS, DATS nor DAS were detectable in urine whereas AMDS was only detectable in the urine samples that were collected after consumption of large amounts (30 g) of garlic. It is possible that this high dosage caused the direct excretion of unmetabolized garlic constituents or that other metabolic pathways are recruited in cases of such high concentrations. On the other hand, the presence of methylmercaptan, allylmercaptan and dimethylsulfide in urine cannot be fully excluded since monitoring of these compounds would require adapted procedures for such highly volatile compounds. In future analyses those compounds should be addressed with methods, that are more specific for highly volatile compounds, like headspace analysis or on-line mass spectrometric volatile monitoring such as proton-transfer reaction mass spectrometry (PTR-MS) [62].

DASO and DASO_2_, compounds which have previously been reported as metabolites of DAS in rat liver, blood and urine [27] were not detectable in any relevant concentration in urine, neither after consumption of 3 g nor of 30 g of raw garlic, in these trials.

### 3.3. Metabolism of Garlic

The main aim of the current study was to identify garlic-derived metabolites in human urine to further elucidate the metabolism of garlic constituents in the human body and to monitor the excretion of different garlic-derived metabolites over a period of up to 26 h. The semi-quantitative analysis revealed high inter-individual variation in the excretion profiles from different volunteers, both with regards to differences in relative substance concentrations and temporal appearance of the compounds’ maxima. The observed differences are related to uptake, distribution, metabolism and excretion of the respective precursors or metabolites, which are strongly individual processes.

The metabolism of garlic components has been addressed in previous studies, although primarily in animal or tissue studies and only few investigations were performed with human subjects. The majority of the human studies have targeted the influence of garlic on human breath composition as mentioned in Section 3.2.

To further clarify the metabolism of garlic compounds, Lawson and Wang [26] investigated the effect of garlic and garlic-derived compounds on breath composition and concluded that garlic compounds that have a dithioallyl group, e.g., DADS and DATS, lead to the formation of AMS, whereas compounds lacking the dithioallyl group have to be metabolized in a different way. As a precursor of AMS they suggested allyl mercaptan, but due to the fast metabolism to AMS they did not expect any accumulation of this compound, AMS itself should be then further oxidized to AMSO and AMSO_2._ Furthermore, they suggested that DAS is metabolized in a different way compared to AMS, due to the absence of the dithioallyl group and proposed DASO_2_ as its metabolite. In our previous study we found AMS, AMSO and AMSO_2_ in human milk [22]. Now, we can confirm the presence of these metabolites in human urine, showing that lactating women and non-lactating people metabolize garlic similarly. However, the presence of DASO_2_ or its presumed precursor DASO could not be confirmed to be present. 

Studies on rat tissues, cells or body fluids offer only disparate data. For example, Sheen et al. [31] treated primary rat hepatocytes with DADS or DAS and observed a conversion of DADS to allyl mercaptan and AMS within 120 min, whereas DAS was only converted to AMS. In the study of Germain et al. [29] DADS was administered directly to rats and the metabolites in the stomach, liver, plasma and urine were monitored over a period of 15 days. Depending on the investigated medium, they observed differences in the metabolite profile. DADS and allyl mercaptan were found in plasma within the first 20 min after oral administration; and AMS, AMSO and AMSO_2_ were detected up to 7 days after oral administration. In the case of rat urine, neither allyl mercaptan nor DADS have been detected, the only metabolites detected were AMS, AMSO and AMSO_2_. In comparison, Brady et al. [27] treated rats with DAS and identified DASO and DASO_2_ as metabolites in liver tissue, blood and urine. In the present study neither of these compounds were detected which is in agreement with the study by Germain et al. [29], who did not observe those metabolites. Instead, they reported AMS, AMSO and AMSO_2_, three metabolites which were now confirmed in human urine after garlic consumption. A possible explanation for these different results is that Brady et al. [27] administered DAS, whereas Germain et al. [29] administered DADS to the rats. It is also possible that DAS is converted to other compounds at a very early stage of digestion, e.g., orally, and can then not be further oxidized to DASO and DASO_2_, whereas DADS may reach the blood and liver and can then be metabolized to AMS, AMSO and AMSO_2_. This mechanism would be in line with the findings of Germain et al. [29], indicating that DADS and allyl mercaptan are precursors of AMS, AMSO and AMSO_2_, at least in rats. However, the authors only observed these compounds in plasma for a short time after administration of DADS and not in urine. Accordingly, it would be necessary to investigate their potential presence in humans not only via urine but also in serum samples.

Regarding the time dependency of the appearance of the metabolites Germain et al. [29] reported the highest concentrations of garlic metabolites (AMS, AMSO and AMSO_2_) in rat plasma and urine 3 days after oral administration of DADS. However, as is discussed in our previous publication on garlic metabolites in human milk [22], the applied amount of this compound is not comparable with the garlic amount consumed by the volunteers in this study. Nevertheless, the temporal appearance of the garlic-derived metabolites in urine between this study and that of Germain et al. is comparable [29]: First, a maximum of the metabolites is observable. In the study by Germain et al. this maximum is followed by a decrease and the formation of a plateau, before it decreased further until the metabolites were no longer measurable. In the present study, a plateau and even a second increase in some cases were observed. According to Germain et al. [29], this pattern is surprising since AMSO and AMSO_2_ are hydrophilic and therefore can be expected to be excreted rapidly in urine. The authors assumed that these metabolites interact with proteins and lipids, leading to a retardation effect in the body, although this would not explain the second increase that we observed for some individuals (e.g., Urine *g*, (Figure 5f)). Another possible explanation could be that garlic constituents are absorbed at different locations throughout the gastrointestinal path, starting from the oral cavity, via the stomach, small intestine and colon; even an interaction with the microbiota of the colon cannot be excluded, as has been reported for other substances such as short-chain fatty acids [63]. Moreover, it is possible that a potential second increase is less pronounced when urine is kept in the bladder for a longer period. Such an effect might further explain the formation of a plateau. 

With regard to potential physiological effects, neither AMS, AMSO nor AMSO_2_ have, to the best of our knowledge, previously been considered as potential active compounds. As such, these should be addressed in future studies. Equally, the identification of conjugated garlic-derived compounds should be subject to further investigation. For these less volatile compounds it is necessary to involve other techniques such as high performance liquid chromatography (HPLC), or to release the respective compounds prior to GC analysis. Possible conjugates are glutathion-conjugates, like S-allylmercaptoglutathion [64], or acetyl-conjugates, like N-acetyl-S-allyl-l-cysteine [65,66]. 

## 4. Materials and Methods 

### 4.1. Chemicals/Materials

The following reference compounds were obtained from the suppliers shown: DMDS, DMTS (Sigma-Aldrich, Steinheim, Germany), AMDS (abcr, Karlsruhe, Germany). The remaining reference substances AMS, AMSO_2_, AMSO, DADS, DAS, DASO_2_, DASO, 3-vinyl-4H-1,2-dithiin and 2-vinyl-4H-1,3-dithiin as well as DATS were synthesized as described in [22].

Dichloromethane (DCM, HPLC grade) and anhydrous sodium sulfate (Na_2_SO_4_) were purchased from VWR (Darmstadt, Germany). The solvent was freshly distilled prior to analysis. 

### 4.2. Human Urine Samples

Urine samples were obtained from 12 volunteers, with one volunteer providing urine on three different days. All volunteers gave written, informed consent to participate in the study and were able to withdraw from participation at any time. The volunteers (age 25–33 years (mean 29); nine females, three males) had no known illnesses that might potentially influence the urine excretion or metabolism at the time of examination. There was no control with regard to the test persons for hormonal contraceptives. The test persons did not take any medication, with the sole exception of one woman who had to use some eye drops because of high intraocular pressure. As the eye drops were applied locally and the expected uptake in the body is very low, this test person was not excluded from the study. 

Each subject was told to refrain from eating food containing high amounts of sulfur compounds for two days prior to the intervention, as well as on the sampling day, in order to eliminate potential sulfurous artefacts in the urine from other dietary sources; foods to be avoided were garlic, onion, ramson, chives, cabbage and leek. Additionally, donors were instructed to keep a record of all foods and beverages consumed during this three-day period. The dietary records are provided in Table S4–S16 of the supplementary material. The procedure followed that of our previous study [22].

On the sampling day volunteers were asked to ingest approx. 3 g raw garlic (equals 1–2 garlic cloves) obtained from a local supermarket. The garlic cloves were peeled and cut into approx. 3 mm cubes using a garlic cutter (Genius GmbH, Limburg/Lahn, Germany). For the high dosage test the volunteer ingested approx. 30 g (1 garlic bulb) of raw garlic. 

The sampling time differed according to the purpose of the test:

- High dosage test: One urine sample was provided 4 min prior to garlic consumption (30 g) and three samples were collected afterwards at approx. 2 h intervals. The urine samples were immediately evaluated and analyzed. This sample set was termed Urine *a*.
- Pre-trials: One urine sample was provided 5 min prior to garlic consumption (3 g) and seven samples were collected afterwards, at 2.1 h, 3.7 h, 6.6 h, 7.8 h, 12.1 h, and 24.1 h after garlic consumption. The urine samples were immediately evaluated and analyzed. This sample set was termed Urine *b*.
- Main tests: In each test set, one urine sample was provided immediately prior to garlic consumption (3 g) and seven samples were collected afterwards, at 0.5 h, 1 h, 2 h, 4 h, 6 h, 8 h and 24 h after garlic consumption. Two volunteers did not provide a sample at 24 h as they were nursing mothers. Another two volunteers each provided an additional sample between 2 h and 4 h after garlic consumption. The samples were immediately evaluated and analyzed or kept frozen at −80 °C until further analysis. This protocol was applied to a set of urine samples from 12 different volunteers that were termed Urine *c* to Urine *n*.

The samples of each set have been termed according to their sampling time. The following list provides the time intervals that are related to the respective sample name:

- *Pre*: prior garlic consumption
- *0.5 h post*: 0.3 h to 0.74 h after garlic consumption
- *1 h post*: 0.75 h to 1.49 h after garlic consumption
- *2 h post*: 1.5 h to 2.49 h after garlic consumption
- *3 h post*: 2.5 h to 3.49 h after garlic consumption
- *4 h post*: 3.5 h to 5 h after garlic consumption
- *6 h post*: 5 h to 7 h after garlic consumption
- *8 h post*: 7 h to 9 h after garlic consumption
- *24 h post*: 23 h to 26 h after garlic consumption

A scheme of the sampling together with the respective methods applied for analysis of the respective samples is provided in the supporting Figure S5. The exact sampling times and the respective volumes of the urine samples are provided in the supplementary Table S3.

### 4.3. Study Design and Ethics Approval

The study was conducted in agreement with the Declaration of Helsinki. Written, informed consent was provided by all 12 volunteers prior to sampling. Withdrawal from the study was possible at any time. The study (registration number 49_13B) was approved by the Ethical Committee of the Medical Faculty, Friedrich-Alexander Universität Erlangen-Nürnberg.

### 4.4. Sensory Analysis

Sensory analyses of the urine samples were carried out by a trained panel (5 to 7 panelists) of the University of Erlangen-Nuremberg (Erlangen, Germany) who exhibited no known illness at the time of examination and with audited olfactory and gustatory function. The panel was trained at recognizing approx. 140 selected odorants at different odorant concentrations according to their odor qualities in preceding weekly training sessions, and in naming these according to an in-house developed flavor language. The panelists were trained for at least 3 months.

For the orthonasal evaluation (smelling) of the urine samples, the samples were presented to the sensory panel in brown glass bottles (capacity 50 mL) in a sensory assessment room at 21 ± 1 °C. No information about the purpose of the experiment was given. The panelists were asked to score the intensities of the attribute “garlic-/cabbage-like” on a scale from 0 (no perception) to 3 (strong perception). The sensory analyses were performed on sample sets Urine *a* to Urine *e*. 

### 4.5. Determination of the Creatinine Concentration in Urine Samples

The creatinine content of each urine sample was determined using a creatinine kit (Labor + Technik Eberhard Lehmann GmbH, Berlin, Germany). This method is based on the Jaffe reaction [67] with creatinine and picric acid forming a yellow complex in an alkaline milieu. The color intensity of the complex is determined photometrically at a wavelength of 492 nm, and is directly proportional to the creatinine concentration. 

### 4.6. Solvent-Assisted Flavor Evaporation (SAFE) of Volatiles from Human Urine

SAFE [68] was used to isolate the volatile compounds from the urine samples. To this aim, 3 to 25 mL DCM were added to the respective volumes of the urine samples, which ranged between 6 to 50 g, respectively, resulting in a ratio of 0.5 mL DCM/1 g urine in each case. Each solution was then stirred for 30 min and immediately underwent SAFE distillation at 50 °C. The resulting distillate was then extracted three times with 25 mL DCM in each case and the combined DCM phases obtained from each sample were dried over anhydrous Na_2_SO_4_ and enriched to a total volume of 100 µL at 50 °C by means of Vigreux distillation and micro-distillation [69]. The final extracts were stored at −80 °C until further analysis. Generally, the urine samples were worked up and measured within a few weeks after collection. 

However, AMS was determined as mentioned below using GC-GC-MS with a specific column configuration of the instrument. This meant that the setup of the instrument had to be changed exclusively for this measurement, so that the samples needed to be analyzed for this specific analyte as a sample set. Because of these analytical requirements, a few samples needed to be stored at −80 °C for a period of up to 12 months for these measurements. To confirm the stability of AMS during storage, a stability test for 1 month was performed. The concentration of AMS remained constant during this period of time confirming that AMS is not degraded in the course of storage. Apart from that, previous studies of our group on the storage of different bodily fluids revealed stability against oxidation at −80 °C for a time period of more than one year [70]. 

### 4.7. High Resolution Gas Chromatography-Olfactometry (HRGC-O)

To identify odor active substances being associated with the garlic/cabbage- like odor of the urine after garlic consumption and to identify other trace metabolites that are odor-active HRGC-O was performed with a Trace Ultra GC (Thermo Finnigan, Dreieich, Germany) using the following capillaries: DB-FFAP (30 m × 0.32 mm, film thickness 0.25 µm; J&W Scientific, Fisons Instruments, Mainz-Kastel, Germany) and DB-5 (30 m × 0.32 mm, film thickness 0.25 µm; J&W Scientific). At the end of the capillaries, the effluent was split between a sniffing port and a flame ionization detector (FID) using two deactivated, uncoated fused silica capillaries (i.D. 0.32 mm). The FID and the sniffing port were held at 250 °C and 270 °C, respectively. The flow rate of the helium carrier gas was 2.0 mL/min. The extract (2 µL) was injected manually into a cold-on-column injector at 40 °C [71], directly on a pre-column of uncoated, deactivated fused silica capillary (2–3 m × 0.32 mm). After 7 min the oven temperature was raised at 8 °C/min to 240 °C (DB-FFAP) or 250 °C (DB-5), which was held for 5 min. In the case of the DB-5 capillary, the oven temperature was further raised to 300 °C at 25 °C/min and held for 5 min. The pre-column was changed regularly to avoid accumulation of any contaminants.

### 4.8. Comparative Aroma Extract Dilution Analysis (cAEDA)

The FD factors of the odorants in human urine before and after garlic consumption were determined by cAEDA [72,73]. The original extracts comprising a total volume of 100 µL were diluted stepwise (1 + 1, v/v) with DCM. HRGC-O analysis was then performed on 2 µL of the original extracts (FD = 1) and the respective dilutions using the DB-5 column until no odor was detectable. The FD factor of each odorant relates to the highest dilution in which this specific odorant could still be perceived. The temperature program for the GC oven was as described above. The odorants were screened by sniffing the effluent after gas chromatographic separation. Linear retention indices (RIs) of the compounds were calculated as described by Van den Dool and Kratz [32]. Complete cAEDAs were performed for Urine *b* to *f*. For Urine *g* to *n* the cAEDA was shortened and only the first 5 min were analyzed by the panelist.

### 4.9. High Resolution Gas Chromatography-Mass Spectrometry (HRGC-MS)

Identification of the metabolites and odor-active derivatives was made based on the mass spectra of the target compounds in the respective urine samples and reference compounds obtained on an Agilent MSD quadrupole system (GC 7890A and MSD 5975C, Agilent Technologies, Waldbronn, Germany) equipped with a Gerstel CIS 4 injection system and GERSTEL MPS 2 autosampler (GERSTEL, Duisburg, Germany). The software used for mass spectral recording and data analysis was MSD ChemStation E.02.00.493 (Agilent Technologies). Analytical capillaries were DB-FFAP and DB-5 (30 m × 0.25 mm, film thickness 0.25 µm, Agilent J&W Scientific, Santa Clara, CA, USA). An uncoated, deactivated fused silica capillary was used as a pre-column (2–3 m × 0.53 mm) and changed regularly to avoid accumulation of impurities. Carrier gas was helium at a total flow of 1.0 mL/min, which was then transferred in un-split mode into the MS using an uncoated, deactivated fused silica capillary (0.3–0.6 m × 0.25 mm) as a transfer line. Identification of garlic-derived metabolites in the urine samples was based on EI mass spectra that were generated in full scan mode (*m*/*z* range 30–350) at 70 eV, whereby representative *m*/*z*-ratios that corresponded to the investigated compounds were extracted (see Table 2). For analysis of AMS, AMSO and AMSO_2_ single ion monitoring (SIM) mode was applied using *m*/*z* 73 + 88, 104 and 120, respectively. The initial temperature of the GC oven was 40 °C, which was held for 7 min and then raised to 240 °C and 250 °C for FFAP and DB-5, respectively, at a rate of 8 °C/min, and held for 7 min. In the case of the DB-5 capillary, the oven temperature was further raised to 300 °C at 25 °C/min and held for 5 min.

### 4.10. Two-Dimensional High Resolution Gas Chromatography-Mass Spectrometry/Olfactometry (HRGC-GC-MS/O) (Heart-Cut)

A two-dimensional gas chromatographic system was used for the mass spectrometric identification of trace constituents. The system consisted of two Varian 450 GCs in combination with a Varian 220 ion trap MS (Varian, Darmstadt, Germany). The first GC was equipped with a multi-column switching system MCS 2 and was connected to the second GC by a cryo-trap system CTS 1 (both: GERSTEL, Duisburg, Germany). A DB-5 column (30 m × 0.32 mm, film thickness 0.25 mm (Agilent J&W Scientific, Santa Clara, CA, USA); first oven) and a DB-FFAP column (30 m × 0.25 mm, film thickness 0.25 mm (Agilent J&W Scientific, Santa Clara, CA, USA); second oven) were used. An uncoated, deactivated fused silica capillary was used as pre-column (2–3 m × 0.53 mm). The flow rate of the helium carrier gas was 2.5 mL/min. In the first oven the effluent was split between an olfaction detection port (ODP, Gerstel) and an FID, as well as a cryo-trap during the cut interval. In the second oven the effluent was transferred to the MS. All split capillaries were made of uncoated, deactivated fused silica material. The FID and the sniffing port were held at 250 °C and 260 °C, respectively. Mass spectra in EI mode were generated at 70 eV ionization energy. The *m*/*z* ranges measured for each compound were as follows: AMS, *m*/*z* 30–100; AMSO and AMSO_2_, *m*/*z* 30–150, AMDS, DADS, DAS, DASO, DASO_2_, DATS, DMDS, DMTS, 2-vinyl-4H-1,3-dithiin and 3-vinyl-4H-1,2-dithiin, *m*/*z* 30–350. The cut time intervals on the main column were determined by injection of the respective reference substances. Application of the samples to the GC system was performed at 40 °C using the cold-on-column technique. The used temperature programs were as follows: For AMS: In the first oven, 40 °C were held for 7 min and then raised to 300 °C at a rate of 20 °C/min. The final temperature was then held for 5 min. The second oven started at a temperature of 40 °C that was held for 7 min, and then raised to 240 °C at a rate of 20 °C/min; the final temperature was held for 5 min. For AMSO/ AMSO_2_ and AMDS, DADS, DAS, DASO, DASO_2_, DATS, DMDS, DMTS, 2-vinyl-4H-1,3-dithiin and 3-vinyl-4H-1,2-dithiin: The first oven started at a temperature of 40 °C. Then, the temperature was raised at a rate of 8 °C/min to 200 °C, and further to 300 °C at a rate of 20 °C/min. The final temperature was held for 5 min. The second oven started at a temperature of 40 °C, and was then heated up to 140 °C at a rate of 20 °C/min. The temperature was further raised to 240 °C at a rate of 8 °C/min, and finally held for 5 min. The transfer line between first and second oven was held at 250 °C and was cooled down to −100 °C for trapping of the respective substances. In the case of DASO, DASO_2_, 2-vinyl-4H-1,3-dithiin and 3-vinyl-4H-1,2-dithiin the transfer line temperature was set to 100 °C and cooled down to −100 °C during trapping.

### 4.11. Identification of Metabolites and Calculation of Metabolite Profiles

Garlic metabolites in human urine were identified by comparing RIs according to Van den Dool and Kratz [32], their odor perceived at the sniffing port via GC-O, and by comparing the EI mass spectra generated by either HRGC-MS or HRGC-GC-MS/O with those of purchased and/or synthesized reference compounds. Comparison of the mass spectra of the analytes with the reference standards was performed with the aid of the NIST Mass Spectral Search Program (Version 2.0 d). The identification was ranked positive if reverse match values were above 900. Two analytical capillaries of different polarities (DB-FFAP and DB-5) were used for calculating the RIs.

To obtain first insights into the time dependency of the metabolite formation and excretion, specifically taking into account potential inter-individual differences, the relative concentration of the metabolites in different urine samples were determined as follows: AMS was determined by HRGC-GC-MS, whereby *m*/*z* 73 and 88 were extracted from the total ion chromatogram and the area of the resulting peak was determined. The concentration was calculated in two different ways. First, the amount was expressed as area/kg. In order to do this, the peak area of AMS was determined, which was then divided by the amount of the investigated urine (in kg). Second, the determined peak area was divided by the amount of the applied human urine (in L), which led to a concentration expressed as area/L. This was then further divided by the creatinine concentration (in mmol/L), so that the final concentration could be expressed as area/mmol creatinine. AMSO and AMSO_2_ were determined by HRGC-MS. For relative quantification a DB-FFAP column was used. For AMSO and AMSO_2_ the SIM mode with *m*/*z* 104 and 120 were applied, respectively. The peak areas were determined and the concentrations were calculated as described for AMS.

## 5. Conclusions 

This study shows that garlic consumption has an effect on the composition of urine. The garlic constituents were shown to be strongly metabolized before being excreted into the urine, with identification of allyl methyl sulfide (AMS), allyl methyl sulfoxide (AMSO) and allyl methyl sulfone (AMSO_2_). Of these compounds only AMS is odorous, but due to the strong inherent smell of urine, the influence of AMS on the overall odor of urine is negligible. 

The metabolites detected were monitored up to 26 h after garlic consumption. The metabolite profiles differed between individuals, resulting in different temporal profiles. In most cases the metabolites reached a maximum concentration between 1 h to 2 h after garlic consumption. Furthermore, a second increase was occasionally observable, about 3 h to 7.8 h after garlic consumption, potentially relating to absorption of garlic derivatives at different locations within the gastrointestinal tract. A range of garlic constituents and metabolites were not detected in urine, indicating that these are metabolized or not excreted in urine, namely DASO, DASO2, DADS, AMDS, DMDS, DMTS, DATS, DAS, 2-vinyl-4H-1,3-dithiin and 3-vinyl-4H-1,2-dithiin. In view of the high inter-individual differences in the metabolite profiles, future studies should address if subject-specific parameters such as gender or hormonal status of the test person have an influence on the metabolite profiles. However, this aspect will require a representative cohort study and quantification of the respective metabolites.

## Figures and Tables

**Table 1:** Flavor dilution (FD) factors obtained for allyl methyl sulfide (AMS) and dimethyl trisulfide (DMTS) in five different urine sample sets as determined via High Resolution Gas Chromatography-Olfactometry (HRGC-O) using a DB-5 capillary. Eight samples per set were collected at different time intervals before and after ingestion of 3 g of raw garlic from different volunteers, whereby urine sets *b* and *f* were provided by the same volunteer, albeit at different days. *Pre* relates to the urine sample that was collected prior to garlic consumption. *0.5 h post* to *24 h post* relate to the urine samples that were obtained after garlic consumption.

Sample	Urine *b*		Urine *c*		Urine *d*		Urine *e*		Urine *f*	
	FD		FD		FD		FD		FD	
	AMS	DMTS	AMS	DMTS	AMS	DMTS	AMS	DMTS	AMS	DMTS
pre	n.d.	n.d.	n.d.	n.d.	n.d.	n.d.	n.d.	4	n.d.	8
0.5 h post	-	-	n.d.	n.d.	32	n.d.	1	1	16	2
1 h post	-	-	n.d.	1	16	1	8	8	8	1
2 h post	2	1	4	1	128	1	16	n.d.	4	4
4 h post	8	2	8	2	4	2	1	1	4	16
6 h post	32	32	16	16	8	4	4	2	64	32
8 h post	8	32	8	4	4	1	4	2	16	4
12 h post	2	4	-	-	-	-	-	-	-	-
24 h post	n.d.	4	n.d.	32	n.d.	1	2	16	n.d.	4

n.d. not detected; - no sample was provided at this time point.

**Table 2:** Compilation of investigated substances, their structure, retention indices (RIs) on DB-FFAP and DB-5 chromatographic capillaries, *m*/*z* ratios used for extracted ion chromatograms/identification via GC-MS, and respective odor qualities. Confirmed presence of the target compounds by means of HRGC-GC-MS analysis is listed for urine sampled after consumption of 30 g (U *a* *4 h post*) and 3 g (U *f 2 h post)* of raw garlic. U *a* was collected at 4.1 h and U *f* at 2 h after garlic consumption. The urine samples were chosen because they contained the highest concentrations of the metabolites AMSO and AMSO_2_, as determined via HRGC-MS.

Substance (Abbreviation)	Structure	RI		*m*/*z*	Odor Quality	Detected in	
		FFAP	DB-5			U *a 4 h post*	U *f 2 h post*
Allyl methyl sulfide (AMS)	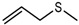	<1000	715	73 + 88	Garlic-like ^a,b^	Yes	Yes
Allyl methyl sulfoxide (AMSO)	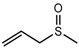	1742	1018	104	Odorless ^c^	Yes	Yes ^d^
Allyl methyl sulfone (AMSO_2_)	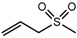	1983	1061	107	Odorless ^c^	Yes	Yes ^d^
Diallyl sulfoxide (DASO)	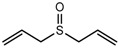	1889	1163	81	Garlic-like ^c^	n.d.	n.a.
Diallyl sulfone (DASO_2_)	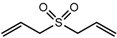	2079	1289	67	Odorless ^c^	n.d.	n.a.
Diallyl disulfide (DADS)	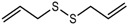	1462	1083	146	Garlic-like ^a^	n.d.	n.a.
					Garlic-like, pungent ^b^		
Allyl methyl disulfide (AMDS)	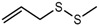	1265	921	120	Garlic-like ^a^	Yes	n.d.
					Cooked garlic-like ^b^		
Dimethyl disulfide (DMDS)	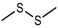	1071	751	94	Cabbage-like ^a^	Yes	n.d. ^e^
					Cooked garlic-like, onion-like, rubber-like ^b^		
Dimethyl trisulfide (DMTS)	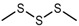	1362	973	126	Garlic-like ^a^	Yes	Yes ^d^
					Burnt garlic-like, diffusive, penetrating, sulfury ^b^		
Diallyl trisulfide (DATS)	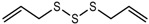	1771	1308	113	Garlic-like ^a^	n.d.	n.a.
					Garlic-like, onion-like ^b^		
Diallyl sulfide (DAS)	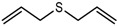	1138	868	114	Garlic-like ^a^	n.d.	n.a.
2-Vinyl-4H-1,3-dithiin	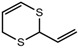	1827	1222	144	Garlic-like ^a^	n.d.	n.a.
3-Vinyl-4H-1,2-dithiin	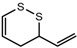	1720	1194	144	Pungent-garlic-like ^a^	n.d.	n.a.

n.d.: not detected. n.a.: not analyzed. Yes: detectable; ^a^ odor quality as described by Tokarska and Karwowska [41]; ^b^ odor quality as described by Ma et al. [42]; ^c^ odor quality as described by Scheffler et al. [22]; ^d^ identified in the sample extracts via HRGC-MS; ^e^ identified in other samples via HRGC-MS.

## Supplementary Materials

The following supplementary material is available online at www.mdpi.com/2218-1989/6/4/43/s1, Table S1: Determined FD factors of AMS in urine sample sets *g* to *n*, Table S2: Compilation of investigated urine samples: time of urine sampling, as well as mass (g), volume (mL) and creatinine content (mmol/L) of the investigated sample; DMTS and DMDS concentrations are given in area/kg and area/mmol creatinine, Table S3: Compilation of investigated urine samples: time of urine sampling, as well as the time of garlic intake, mass (g), volume (mL) and creatinine content (mmol/L) of the investigated sample; AMS, AMSO and AMSO_2_ concentrations are given in area/kg and area/mmol creatinine, Tables S4–S16: dietary records—test person *b* to *n*, Figure S1: Perceived intensity of a “garlic-/cabbage-like” odor in urine at different time intervals before and after ingestion of raw garlic, Figure S2: Time-resolved metabolite profiles of AMS, AMSO and AMSO_2_ for urine set *a*, Figure S3: Time-resolved profiles of DMTS excretion in urine for sets *b* to *n*, Figure S4: Time-resolved profiles of DMDS excretion in urine for sets *b* to *n*, Figure S5: Scheme of urine sampling and analytical methods.

## Abbreviations

The following abbreviations are used in this manuscript:

AMDS:	Allyl methyl disulfide
AMS:	Allyl methyl sulfide
AMSO:	Allyl methyl sulfoxide
AMSO_2_:	Allyl methyl sulfone
cAEDA:	Comparative aroma extract dilution analysis
CVD:	Cardiovascular disease
DADS:	Diallyl disulfide
DAS:	Diallyl sulfide
DASO:	Diallyl sulfoxide
DASO_2_:	Diallyl sulfone
DATS:	Diallyl trisulfide
DMDS:	Dimethyl disulfide
DMTS:	Dimethyl trisulfide
FD factor:	Flavor dilution factor
FID:	Flame ionization detector
HDL:	High density lipoprotein
HRGC-GC-MS:	Two dimensional high-resolution gas chromatography-mass spectrometry
HRGC-MS:	High-resolution gas chromatography-mass spectrometry
HRGC-O:	High-resolution gas chromatography-olfactometry
LDL:	Low density lipoprotein
RI:	(Linear) retention indices
SAFE:	Solvent-assisted flavor evaporation
TC:	Total cholesterol
TIC:	Total ion chromatogram
