# Supplementary material for: Detection of Volatile Metabolites Derived from Garlic (Allium sativum) in Human Urine
Source: Metabolites. 2016 Dec 1;6(4):43. doi: 10.3390/metabo6040043 (PMC5192449; doi:10.3390/metabo6040043)
Supplement: Supplementary file 1 [file metabolites-06-00043-s001.pdf]

Supplementary Materials: Detection of Volatile Metabolites Derived from Garlic (*Allium sativum*) in Human Urine

Laura Scheffler, Yvonne Sauermann, Anja Heinlein, Constanze Sharapa and Andrea Buettner

Table S1. Determined flavor dilution (FD) factors of AMS in different urine sample sets g to n as determined via HRGC-O using a DB-5 capillary. Six to nine samples per set were collected at different time intervals before and after ingestion of 3 g of raw garlic from different volunteers. "*Pre*" relates to the urine sample that was collected prior to garlic consumption. "*0.5 h post*" to "*24 h post*" relate to the urine samples that were obtained after garlic consumption.

sample	Urine g	Urine h	Urine i	Urine j	Urine k	Urine l	Urine m	Urine n
pre	n.d.	n.d.	n.d.	n.d.	n.d.	n.d.	n.d.	n.d.
0.5 h post	8	4	8	4	8	8	8	8
1 h post	32	8	32	16	8	8	8	8
2 h post	8	8	16	8	16	8	-	16
3 h post	-	-	-	-	4	8	32	-
4 h post	8	8	16	4	16	8	32	16
6 h post	8	8	16	2	8	8	32	n.d.
8 h post	n.d.	8	16	n.d.	16	4	-	-
24 h post	n.d.	2	n.d.	n.d.	n.d.	n.d.	-	-

n.d. not detected; - no sample was provided at this time point.

Table S2. Compilation of investigated urine samples: time of urine sampling, as well as mass (g), volume (mL) and creatinine content (mmol/L) of the investigated sample; DMTS and DMDS-concentrations are given in area/kg and area/mmol creatinine.

	Sample	Time (h)	Mass (g)	Volume (mL)	Creatinine (mmol/L)	DMTS		DMDS	
						(area/kg)	(area/mmol creatinine)	(area/kg)	(area/mmol creatinine)
urine a	*pre*	0.00	53.1486			5926.8		0.0	
	2 h post	2.17	50.1150			5547.2		18497.5	
	4 h post	4.08	49.5297			9408.5		1221872.9	
	6 h post	5.83	55.6357			21874.4		73100.5	
urine b	*pre*	0.00	18.2110		1.67	0.0	0.0 *	110043.4	65894.2 *
	2 h post	2.08	26.0446		1.75	0.0	0.0 *	68689.9	39251.4 *
	4 h post	3.67	37.1800		1.47	28294.8	19248.2 *	467670.8	318143.4 *
	6 h post	6.58	54.3923		2.62	315154.9	120288.1 *	3538791.3	1350683.7 *
	8 h post	7.75	49.5103		1.47	82346.5	56018.0 *	251624.4	171173.1 *
	12 h post	12.12	61.7643		3.91	9973.4	2550.7 *	326078.3	83396.0 *
	21 h post	21.08	38.1209		9.89	106214.7	10739.6 *	723461.4	73150.8 *
	24 h post	24.08	50.4667		3.08	19577.3	6356.3 *	671868.8	218139.2 *
urine c	*pre*	0.00	39.6450	39.2	5.96	30319.1	5143.4	198713.6	33710.1
	0.5 h post	0.63	49.9958	50.0	1.11	7280.6	6544.4	110969.3	99747.5
	1 h post	1.08	19.9021	50.0	0.57	158224.5	110309.7	284844.3	198585.4
	2 h post	1.95	49.9436	50.0	0.66	11873.4	18038.0	179282.2	272365.1
	4 h post	3.78	50.3893	50.0	2.60	21492.7	8340.9	149496.0	58016.5
	6 h post	5.75	50.5265	50.0	3.88	79582.0	20723.9	304652.0	79334.4
	8 h post	8.13	50.2804	50.0	3.13	45007.6	14438.7	199501.2	64001.1
	24 h post	24.15	50.743	50.0	7.31	122066.1	16955.5	479711.5	66634.1

Table S2. *Cont.*

	Sample	Time (h)	Mass (g)	Volume (mL)	Creatinine (mmol/L)	DMTS		DMDS	
						(area/kg)	(area/mmol creatinine)	(area/kg)	(area/mmol creatinine)
urine d	*pre*	0.00	50.1570	50.0	3.81	15331.9	4034.1	45596.8	11997.5
	0.5 h post	0.47	49.8599	50.0	1.13	7380.7	6498.8	36141.3	31823.0
	1 h post	1.05	49.9479	50.0	1.63	11532.0	7067.9	42364.1	25964.7
	2 h post	2.13	49.9834	50.0	1.74	6082.0	3493.4	21607.2	12410.7
	4 h post	4.00	50.5099	50.0	5.64	30964.2	5550.7	276322.1	49533.6
	6 h post	5.75	50.5739	50.0	5.17	92498.3	18110.6	270495.3	52961.3
	8 h post	7.52	50.0155	50.0	1.82	15475.2	8492.0	78395.7	43019.4
	24 h post	24.92	50.019	50.0	2.40	19052.8	7929.7	113396.9	47195.5
urine e	*pre*	0.00	50.0938	50.0	0.93	15251.4	16346.5	38607.6	41379.8
	0.5 h post	0.77	49.9007	50.0	0.85	17154.1	20161.1	7815.5	9185.5
	1 h post	1.45	49.8642	50.0	0.74	17627.9	23887.8	11531.3	15626.3
	2 h post	2.25	49.9592	50.0	1.39	0.0	0.0	6585.4	4746.5
	4 h post	3.88	50.0838	50.0	2.04	2775.3	1364.1	20665.4	10157.1
	6 h post	5.72	50.0233	50.0	1.81	5017.7	2773.2	42900.0	23710.3
	8 h post	7.88	50.2255	50.0	3.09	5077.1	1652.6	45494.8	14809.0
	24 h post	24.68	50.0227	50.0	1.78	40801.5	22917.1	121504.8	68246.1
urine f	*pre*	0.00	32.5189	32.0	14.31	24693.3	1753.0	202005.6	14340.7
	0.5 h post	0.55	49.9647	50.0	2.99	0.0	0.0	48534.3	16193.9
	1 h post	1.03	49.8430	50.0	0.95	0.0	0.0	0.0	0.0
	2 h post	2.03	49.9875	50.0	1.74	0.0	0.0	0.0	0.0
	4 h post	4.07	50.0798	50.0	3.92	57528.2	14690.2	94848.6	24220.3
	6 h post	6.02	50.3908	50.0	6.78	146296.5	21756.1	379533.6	56441.2
	8 h post	8.05	50.503	50.0	6.94	0.0	0.0	0.0	0.0
	24 h post	24.68	39.7167	39.0	14.10	0.0	0.0	33512.4	2421.0
urine g	*pre*	0.00	50.1354	50.0	4.31	19128.2	4450.3	124363.2	28934.1
	0.5 h post	1.12	49.8514	50.0	1.18	5917.6	4989.2	33860.6	28548.6
	1 h post	1.73	49.9335	50.0	1.22	4846.4	3951.9	24272.3	19792.2
	2 h post	2.92	50.0587	50.0	3.96	60988.4	15433.2	0.0	0.0
	4 h post	4.67	49.9454	50.0	2.47	33296.4	13480.8	0.0	0.0
	6 h post	6.92	50.8932	50.0	12.82	289331.4	22977.5	686751.9	54539.0
	8 h post	7.83	6.8311	6.0	15.86	0.0	0.0	0.0	0.0
	24 h post	24.53	18.411	18.0	18.94	0.0	0.0	0.0	0.0
urine h	*pre*	0.00	10.1392	10.0	15.17	44086.3	2947.1	890405.6	59522.8
	0.5 h post	1.02	49.9072	50.0	1.05	44342.3	42111.3	0.0	0.0
	1 h post	1.73	49.9145	50.0	0.75	67555.5	90030.4	0.0	0.0
	2 h post	2.92	49.8837	50.0	0.84	0.0	0.0	0.0	0.0
	4 h post	4.82	49.7932	50.0	0.94	0.0	0.0	0.0	0.0
	6 h post	6.92	49.798	50.0	1.49	7630.8	5112.9	0.0	0.0
	8 h post	7.83	50.1232	50.0	6.10	16479.4	2709.7	0.0	0.0
	24 h post	23.63	50.024	50.0	2.08	32784.3	15778.2	0.0	0.0
urine i	*pre*	0.00	19.3317	19.0	22.94	19656.8	871.8	87524.6	3881.8
	0.5 h post	0.92	34.5982	34.0	19.15	22631.2	1202.3	130324.7	6923.5
	1 h post	1.50	26.2297	25.8	14.51	53069.6	3717.4	204729.8	14341.0
	2 h post	2.73	35.5222	35.0	12.07	43775.4	3680.6	107285.0	9020.5
	4 h post	4.62	33.5814	33.0	12.31	29480.6	2436.4	120870.5	9989.4
	6 h post	6.67	50.6976	50.0	9.10	19843.1	2209.9	95507.5	10636.4
	8 h post	7.45	43.5456	43.0	10.10	16810.0	1685.0	38442.5	3853.5
	24 h post	23.88	15.5162	15.2	37.11	63546.5	1748.0	163313.2	4492.4

Table S2. *Cont.*

	Sample	Time (h)	Mass (g)	Volume (mL)	Creatinine (mmol/L)	DMTS		DMDS	
						(area/kg)	(area/mmol creatinine)	(area/kg)	(area/mmol creatinine)
urine j	*pre*	0.00	50.7676	50.0	11.79	12409.5	1068.8	76564.6	6594.2
	0.5 h post	0.53	49.9728	50.0	0.80	0.0	0.0	9465.1	11755.4
	1 h post	1.03	49.9724	50.0	0.66	16088.9	24442.0	10265.7	15595.4
	2 h post	2.07	50.0155	50.0	1.37	0.0	0.0	13535.8	9850.8
	4 h post	4.12	40.2985	40.0	7.45	15509.3	2096.2	33524.8	4531.1
	6 h post	6.03	50.1150	50.0	2.76	0.0	0.0	0.0	0.0
	8 h post	7.78	50.0429	50.0	1.17	0.0	0.0	8952.3	7626.9
	24 h post	24.12	45.8566	45.0	20.84	35087.6	1715.6	135683.8	6634.4
urine k	*pre*	0.00	50.0446	50.0	5.75	5475.1	952.3	41083.4	7146.0
	0.5 h post	0.77	50.0027	50.0	2.29	2319.9	1013.1	7739.6	3379.8
	1 h post	1.30	49.9764	50.0	3.07	3421.6	1113.2	4202.0	1367.1
	2 h post	2.33	49.9929	50.0	2.96	3220.5	1086.5	17262.5	5823.8
	3 h post	3.35	49.9729	50.0	2.90	0.0	0.0	32117.4	11054.2
	4 h post	4.38	50.0049	50.0	5.03	0.0	0.0	47935.3	9536.3
	6 h post	6.22	49.9742	50.0	5.08	0.0	0.0	5422.8	1066.1
	8 h post	7.92	50.2461	50.0	11.01	17653.1	1611.8	49794.9	4546.4
	24 h post	23.80	50.2049	50.0	7.43	0.0	0.0	22069.6	2982.3
urine l	*pre*	0.00	51.2038	50.0	41.00	17811.2	444.9	151883.3	3793.7
	0.5 h post	0.75	39.7135	39.0	25.03	51393.1	2090.8	224759.8	9143.7
	1 h post	1.30	50.0618	50.0	4.09	0.0	0.0	57828.5	14159.8
	2 h post	2.33	50.0743	50.0	5.47	12860.9	2354.6	92262.9	16891.6
	3 h post	3.12	50.0046	50.0	3.32	2819.7	850.6	30177.2	9103.6
	4 h post	4.33	50.2867	50.0	12.32	15590.6	1272.5	212123.7	17314.0
	6 h post	6.20	50.8602	50.0	27.19	36531.5	1366.9	175382.7	6562.1
	8 h post	7.90	50.9033	50.0	23.54	22513.3	973.8	126593.0	5475.5
	24 h post	26.05	30.5201	30.0	28.57	21821.7	777.1	36664.4	1305.7
urine m	*pre*	0.00	50.0039	50.0	2.45	11839.1	4841.5	75174.1	30741.6
	0.5 h post	0.77	49.9662	50.0	1.80	10166.9	5636.6	132809.8	73630.6
	1 h post	1.42	49.8828	50.0	1.53	6856.1	4479.5	126416.3	82595.7
	3 h post	2.67	50.3904	50.0	5.22	53204.6	10264.4	479456.4	92497.9
	4 h post	3.75	40.5086	40.0	7.81	79785.5	10342.1	375821.4	48715.5
	6 h post	5.92	50.7212	50.0	10.27	24644.5	2434.4	181502.0	17929.1
urine n	*pre*	0.00	35.7715	35.0	16.70	39388.9	2410.3	71537.4	4377.6
	0.5 h post	1.00	30.6561	30.0	13.53	22997.1	1736.8	51148.1	3862.9
	1 h post	1.50	30.0953	30.0	2.60	22927.2	8838.1	41136.0	15857.3
	2 h post	2.67	50.1476	50.0	3.68	7218.7	1969.0	22732.9	6200.9
	4 h post	4.37	45.8559	45.0	16.86	27193.9	1643.2	139022.5	8400.5
	6 h post	6.50	37.8167	37.0	19.77	55055.0	2846.6	90832.9	4696.5

* Calculated under the assumption 1 mL urine equals 1 g urine.

Table S3. Compilation of investigated urine samples: time of urine sampling, as well as the time of garlic intake, mass (g), volume (mL) and creatinine content (mmol/L) of the investigated sample; AMS, AMSO and $AMSO_2$-concentrations are given in area/kg and area/mmol creatinine.

	Sample	Time (h)	Mass (g)	Volume (mL)	Creatinine (mmol/L)	AMS		AMSO		$AMSO_2$	
						(area/kg)	(area/mmol creatinine)	(area/kg)	(area/mmol creatinine)	(area/kg)	(area/mmol creatinine)
urine a	*pre*	0.00	53.1486			2709		0		0	
	2 h post	2.17	50.1150			551851		147868143		25609638	
	4 h post	4.08	49.5297			731844		198612731		45229428	
	6 h post	5.83	55.6357			829449		196025448		67629166	
	garlic intake	0.07									
urine b	*pre*	0.00	18.2110		1.67	0	0.0 *	0	0.0 *	0	0.0 *
	2 h post	2.08	26.0446		1.75	25264	14436.8 *	61396451	35083686.1 *	11712793	6693024.4 *
	4 h post	3.67	37.1800		1.47	19392	13191.9 *	27941366	19007732.2 *	14414578	9805835.2 *
	6 h post	6.58	54.3923		2.62	93910	35843.6 *	8602118	3283251.3 *	9027068	3445445.9 *
	8 h post	7.75	49.5103		1.47	13997	9521.8 *	4866078	3310257.4 *	6382248	4341665.1 *
	12 h post	12.12	61.7643		3.91	10265	2625.3 *	5232359	1338199.3 *	6228614	1592996.0 *
	21 h post	21.08	38.1209		9.89	12880	1302.3 *	5027846	508376.7 *	3408944	344685.9 *
	24 h post	24.08	50.4667		3.08	3864	1254.5 *	2295236	745206.6 *	1835547	595956.8 *
	garlic intake	0.08									
urine c	*pre*	0.00	39.6450	39.2	5.96	0	0.0	0	0.0	0	0.0
	0.5 h post	0.63	49.9958	50.0	1.11	8841	7946.7	13939734	12794397.3	1028266	924282.6
	1 h post	1.08	19.9021	50.0	0.57	54768	38182.8	78210046	54717046.1	13358942	9313476.9
	2 h post	1.95	49.9436	50.0	0.66	37242	56578.0	33506577	51392272.9	8811780	13386832.4
	4 h post	3.78	50.3893	50.0	2.60	35642	13832.2	30865036	12057294.2	8560686	3322235.2
	6 h post	5.75	50.5265	50.0	3.88	28777	7493.8	14106376	3706338.4	8790892	2289234.0
	8 h post	8.13	50.2804	50.0	3.13	14678	4708.7	6165683	2032776.7	5572370	1787646.7
	24 h post	24.15	50.743	50.0	7.31	788	109.5	246031	23033.7	249236	34620.1
	garlic intake	0.17									
urine d	*pre*	0.00	50.1570	50.0	3.81	0	0.0	0	0.0	0	0.0
	0.5 h post	0.47	49.8599	50.0	1.13	24388	21474.4	13266880	11850649.6	1336284	1176622.7
	1 h post	1.05	49.9479	50.0	1.63	88272	54101.4	32913660	20268289.9	5356822	3283165.1
	2 h post	2.13	49.9834	50.0	1.74	71864	41277.1	29935489	17279463.7	7224679	4149700.6
	4 h post	4.00	50.5099	50.0	5.64	84597	15165.0	32655112	5830828.4	12151281	2178245.2
	6 h post	5.75	50.5739	50.0	5.17	79764	15617.4	20506070	3996540.9	12698922	2486370.4
	8 h post	7.52	50.0155	50.0	1.82	26292	14427.6	5440388	3063540.1	6122842	3359887.0
	24 h post	24.92	50.019	50.0	2.40	1799	748.9	380770	200357.2	929667	386924.6
	garlic intake	0.10									

Table S3. *Cont.*

	Sample	Time (h)	Mass (g)	Volume (mL)	Creatinine (mmol/L)	AMS		AMSO		$AMSO_2$	
						(area/kg)	(area/mmol creatinine)	(area/kg)	(area/kg)	(area/mmol creatinine)	(area/kg)
urine e	*pre*	0.00	50.0938	50.0	0.93	0	0.0	0	0.0	0.0	0.0
	0.5 h post	0.77	49.9007	50.0	0.85	8236	9680.1	5889981	6930051.2	648978.4	767283.4
	1 h post	1.45	49.8642	50.0	0.74	35196	47694.1	12127876	16455469.6	2488620.7	3384807.7
	2 h post	2.25	49.9592	50.0	1.39	47138	33975.7	21495418	15467377.4	5969869.6	4287459.3
	4 h post	3.88	50.0838	50.0	2.04	34782	17095.3	9634387	4692755.1	4455042.1	2164191.3
	6 h post	5.72	50.0233	50.0	1.81	24309	13435.1	5627221	3072008.9	4064367.9	2223535.7
	8 h post	7.88	50.2255	50.0	3.09	15291	4977.4	3488713	1080858.1	3690340.9	1168479.4
	24 h post	24.68	50.0227	50.0	1.78	3498	1965.0	806598	415620.8	723893.8	384198.8
	garlic intake	0.22									
urine f	*pre*	0.00	32.5189	32.0	14.31	0	0.0	0	0.0	0.0	0.0
	0.5 h post	0.55	49.9647	50.0	2.99	30802	10277.3	14706735	4921190.4	1701715.7	583577.4
	1 h post	1.03	49.8430	50.0	0.95	49696	52032.3	16496262	17324329.0	3297595.0	3511266.9
	2 h post	2.03	49.9875	50.0	1.74	63936	36717.9	17517307	10087145.2	4892169.9	2839775.1
	4 h post	4.07	50.0798	50.0	3.92	48523	12390.6	13514300	3460899.1	5476275.8	1409483.9
	6 h post	6.02	50.3908	50.0	6.78	91386	13590.2	9812221	1463380.8	5838786.3	872968.2
	8 h post	8.05	50.503	50.0	6.94	27880	4058.4	4026823	590201.1	3572983.2	524601.1
	24 h post	24.68	39.7167	39.0	14.10	1586	114.6	408252	29560.2	323730.3	23462.0
	garlic intake	0.05									
urine g	*pre*	0.00	50.1354	50.0	4.31	1636	380.5	14410	15880.1	63747.4	14831.3
	0.5 h post	1.12	49.8514	50.0	1.18	23149	19517.2	3683963	3151426.1	885531.8	746610.7
	1 h post	1.73	49.9335	50.0	1.22	34306	27973.6	11226718	9198438.4	3420148.8	2788870.3
	2 h post	2.92	50.0587	50.0	3.96	47924	12127.1	11784496	2995700.1	4307922.5	1090122.6
	4 h post	4.67	49.9454	50.0	2.47	51616	20898.1	6075067	2481436.6	4576797.9	1853025.9
	6 h post	6.92	50.8932	50.0	12.82	28412	2256.4	3910436	314827.2	3503945.5	278269.1
	8 h post	7.83	6.8311	6.0	15.86	7905	567.4	2665482	195202.3	2105224.6	151119.9
	24 h post	24.53	18.411	18.0	18.94	4508	243.5	520974	31044.2	341915.2	18465.7
	garlic intake	0.63									
urine h	*pre*	0.00	10.1392	10.0	15.17	2564	171.4	232568	19146.5	176345.3	11788.5
	0.5 h post	1.02	49.9072	50.0	1.05	6071	5765.8	3604725	3474499.9	415731.6	394815.3
	1 h post	1.73	49.9145	50.0	0.75	14865	19811.0	9682283	12975223.1	1332378.4	1775644.8
	2 h post	2.92	49.8837	50.0	0.84	28787	34112.2	11975876	14255060.4	2540328.8	3010256.0
	4 h post	4.82	49.7932	50.0	0.94	19722	20878.8	7976307	8501330.9	2588184.7	2740049.4
	6 h post	6.92	49.798	50.0	1.49	14378	9633.8	6580397	4445166.9	3132274.4	2098729.9
	8 h post	7.83	50.1232	50.0	6.10	18614	3060.7	6264646	1038932.4	3524535.5	579529.8
	24 h post	23.63	50.024	50.0	2.08	2539	1221.8	297366	169028.6	330961.1	159282.7
	garlic intake	0.50									

Table S3. *Cont.*

	Sample	Time (h)	Mass (g)	Volume (mL)	Creatinine (mmol/L)	AMS		AMSO		$AMSO_2$	
						(area/kg)	(area/mmol creatinine)	(area/kg)	(area/kg)	(area/mmol creatinine)	(area/kg)
urine i	*pre*	0.00	19.3317	19.0	22.94	0	0.0	0	0.0	0.0	0.0
	0.5 h post	0.92	34.5982	34.0	19.15	9740	517.5	21225792	1128422.2	2443216.7	130683.6
	1 h post	1.50	26.2297	25.8	14.51	49982	3501.1	31358773	2198958.6	6279361.5	442456.5
	2 h post	2.73	35.5222	35.0	12.07	40425	3399.0	24635876	2074977.7	6867832.1	581461.5
	4 h post	4.62	33.5814	33.0	12.31	52350	4326.5	20143541	1668248.5	8155278.7	677870.6
	6 h post	6.67	50.6976	50.0	9.10	41501	4621.8	9715785	1088086.4	5762099.5	648480.6
	8 h post	7.45	43.5456	43.0	10.10	11069	1109.5	4642071	470382.6	4112459.6	417881.3
	24 h post	23.88	15.5162	15.2	37.11	1418	39.0	1092252	28519.9	881379.5	22542.4
	garlic intake	0.08									
urine j	*pre*	0.00	50.7676	50.0	11.79	0	0.0	0	0.0	0.0	0.0
	0.5 h post	0.53	49.9728	50.0	0.80	11206	13917.6	4088646	5198776.8	1153147.3	1432173.8
	1 h post	1.03	49.9724	50.0	0.66	10466	15899.4	5989328	9248660.7	2506143.4	3807289.8
	2 h post	2.07	50.0155	50.0	1.37	13576	9879.9	4824676	3578254.0	2489448.3	1811714.2
	4 h post	4.12	40.2985	40.0	7.45	3499	472.9	2115344	291025.6	1717830.7	232175.0
	6 h post	6.03	50.1150	50.0	2.76	3033	1101.1	801620	319972.0	1089214.8	395440.6
	8 h post	7.78	50.0429	50.0	1.17	1099	936.3	367799	393383.5	548749.2	467502.8
	24 h post	24.12	45.8566	45.0	20.84	785	38.4	91073	561.9	53056.7	2594.3
	garlic intake	0.03									
urine k	*pre*	0.00	50.0446	50.0	5.75	0	0.0	0	0.0	0.0	0.0
	0.5 h post	0.77	50.0027	50.0	2.29	25779	11257.4	5505849	2412621.0	820255.7	358201.0
	1 h post	1.30	49.9764	50.0	3.07	39779	12941.5	14055977	4577664.8	2847003.8	926232.2
	2 h post	2.33	49.9929	50.0	2.96	37525	12659.7	12987337	4386606.2	3915536.0	1320965.7
	3 h post	3.35	49.9729	50.0	2.90	22612	7782.7	7711865	2659616.2	4080411.6	1404397.3
	4 h post	4.38	50.0049	50.0	5.03	29777	5923.9	6405217	1275045.9	3757291.8	747479.8
	6 h post	6.22	49.9742	50.0	5.08	15528	3052.8	5117965	1006917.4	4173993.8	820618.6
	8 h post	7.92	50.2461	50.0	11.01	37416	3416.2	3547811	321331.4	3367962.9	307504.9
	24 h post	23.80	50.2049	50.0	7.43	2530	341.8	391873	51736.4	327916.2	44312.0
	garlic intake	0.13									
urine l	*pre*	0.00	51.2038	50.0	41.00	0	0.0	0	0.0	0.0	0.0
	0.5 h post	0.75	39.7135	39.0	25.03	18457	750.9	10443854	424876.1	1156231.5	47037.7
	1 h post	1.30	50.0618	50.0	4.09	16200	3966.7	6633361	1624234.7	1687354.4	413163.0
	2 h post	2.33	50.0743	50.0	5.47	13540	2478.9	5214431	954662.8	2008335.6	367687.9
	3 h post	3.12	50.0046	50.0	3.32	8779	2648.4	3347492	1009846.4	1837171.0	554224.0
	4 h post	4.33	50.2867	50.0	12.32	20264	1654.0	2805553	228994.8	1668572.4	136192.2
	6 h post	6.20	50.8602	50.0	27.19	31616	1182.9	2349892	87923.5	1737822.5	65022.3
	8 h post	7.90	50.9033	50.0	23.54	8035	347.5	1483460	64163.7	1323057.6	57225.8
	24 h post	26.05	30.5201	30.0	28.57	0	0.0	144298	5138.7	126506.8	4505.1
	garlic intake	0.13									

Table S3. *Cont.*

	Sample	Time (h)	Mass (g)	Volume (mL)	Creatinine (mmol/L)	AMS		AMSO		$AMSO_2$	
						(area/kg)	(area/mmol creatinine)	(area/kg)	(area/kg)	(area/mmol creatinine)	(area/kg)
urine m	*pre*	0.00	50.0039	50.0	2.45	0	0.0	0	0.0	0.0	0.0
	0.5 h post	0.77	49.9662	50.0	1.80	26798	14857.1	9463304	5251620.4	1499653.8	831417.9
	1 h post	1.42	49.8828	50.0	1.53	26983	17629.8	9136842	5978242.4	2256268.7	1474161.1
	3 h post	2.67	50.3904	50.0	5.22	31315	6041.5	9744504	1872355.3	2856119.4	551009.3
	4 h post	3.75	40.5086	40.0	7.81	19280	2499.1	7362093	944506.8	3156391.5	409144.3
	6 h post	5.92	50.7212	50.0	10.27	28450	2810.3	4316870	415547.9	2930037.1	289433.7
	garlic intake	0.08									
urine n	*pre*	0.00	35.7715	35.0	16.70	4389	268.6	958473	44307.0	1004402.9	61462.0
	0.5 h post	1.00	30.6561	30.0	13.53	9231	697.2	7076406	534442.5	1336471.4	100936.4
	1 h post	1.50	30.0953	30.0	2.60	23027	8876.5	26998435	10407496.1	4058241.7	1564391.9
	2 h post	2.67	50.1476	50.0	3.68	54399	14838.6	18852767	5142488.3	4426971.6	1207549.5
	4 h post	4.37	45.8559	45.0	16.86	77177	4663.4	12180875	736032.5	4719392.7	285170.5
	6 h post	6.50	37.8167	37.0	19.77	21525	1112.9	5720991	295801.4	3612240.1	186769.3
	garlic intake	0.50									

* calculated under the assumption 1 mL urine equals 1 g urine.

Table S4. Dietary record—*test person b*.

		Food	Beverages
DAY 1	break-fast	porridge with coconut blossom sugar	coffee, juice
	S1		
	lunch	pizza with salami and ham	water, cola, coffee
	S2		
	dinner	croissants	juice
	S3		
DAY 2	break-fast		coffee, juice
	S1		
	lunch	pizza with salami and ham	water
	S2		
	dinner	pasta with pepper, pesto, cream	tea with honey
	S3		
SAMPLING DAY	break-fast	"Alnatura Frühstücksbrei" (porridge, spelt, rice (puffed), linseed, different nuts)	coffee, water
	S1		
	lunch	panini with tomato and mozzarella	tea
	S2		
	dinner	pasta with cream sauce	water, tea
	S3		

S1, S2, S3: Snacks.

Table S5. Dietary record—*test person c*.

		Food	Beverages
DAY 1	break-fast	breakfast cereals	milk
	S1	cookies	tea
	lunch	pretzel, roll with turkey, cheese and salad	
	S2	apple, cake	
	dinner	rice with tomato, egg	
	S3		
DAY 2	break-fast	toast	tea
	S1	cookies	
	lunch	roll with ham, cucumber, orange	tea
	S2	apple, cookies	
	dinner	salad, cutlet, orange	
	S3		
SAMPLING DAY	break-fast	toast	milky coffee
	S1	cookies	
	lunch	salad (ewe's cheese, olives), bread	
	S2	muffin	
	dinner	cutlet, egg, orange	
	S3		

S1, S2, S3: Snacks.

Table S6. Dietary record—*test person d*.

		Food	Beverages
DAY 1	break-fast		water
	S1		
	lunch	roll with mild, full-fat cheese, tomato	water
	S2	2 truffels	
	dinner	scrampled eggs with pepper and basil, 2 slices of sunflower-bread	water, orange spritzer, Fanta classic
	S3	apple cake	
DAY 2	break-fast		water
	S1		
	lunch	bagel with cream cheese and rocket, orange, chocolate bar with milk-filling (Kinderriegel)	water
	S2	spelt-chocolate-cookie	
	dinner	pizza	water, orange spritzer
	S3		
SAMPLING DAY	break-fast	chocolate-cereal	water, apple spritzer
	S1		
	lunch	turkey-egg-round flat bread	water, apple spritzer
	S2	rhubarb-pie	
	dinner	pasta with ham	water, lemonade
	S3		

S1, S2, S3: Snacks.

Table S7. Dietary record—*test person e*.

		Food	Beverages
DAY 1	break-fast	cereal	coffee
	S1		
	lunch	bratwurst, mustard, horseradish	water
	S2		
	dinner	pasta with salmon, tomato and escallion	water
	S3	pancake with chocolate, banana	
DAY 2	break-fast	chocolate muffin, half and half cookie	coffee
	S1		
	lunch		water, cola
	S2		
	dinner	pancake, omelet with pepper	cacao
	S3		
SAMPLING DAY	break-fast	peach, cereal	coffee
	S1		
	lunch	panini with tomato and mozzarella, granary bread with cream cheese	water
	S2		
	dinner	pasta with vegetables, soy cream	water
	S3		

S1, S2, S3: Snacks.

Table S8. Dietary record—*test person f.*

		Food	Beverages
DAY 1	break-fast	bread with Nutella, tea sausage spread<br>chocolate-cream-dessert<br>rockmelon	cappuccino, water, orange juice
	S1		
	lunch	naan bread au naturel with cheese, ham and rocket<br>bread with sausage, meat salad	orange spritzer
	S2	nut roll	coffee with milk
	dinner	bread with meat salad, sausage, cheese	Iso grapefruit (lemonade), water
	S3	chocolate-oat-cookie	
DAY 2	break-fast	toast with cheese, ham, jam, Nutella	coffee, orange spritzer
	S1	chocolate-oat-cookie	water
	lunch	-	
	S2		
	dinner	sushi (artichoke, salmon, cucumber, carrot, sprouts, peanut), wasabi, ginger, soy sauce	orange spritzer
	S3	brownie with vanilla ice-cream	cider, Guiness, water
SAMPLING DAY	break-fast	(after garlic-intake)<br>prezel with pumpkin seeds,<br>croissante with nut-nougat croissante	coffee with milk, water
	S1		water
	lunch	sandwich tomato/mozzarella<br>sushi with smoked salmon	coffee with milk, water
	S2	donuts (raspberry, chocolate, nut)	coffee
	dinner	sandwich with cheese, rocket, bacon	water, passion fruit spritzer
	S3	cheeseburger, chickenburger (McDonalds)	water

S1, S2, S3: Snacks.

Table S9. Dietary record—*test person g*.

		Food	Beverages
DAY 1	break-f ast	-	coffee
	S1		
	lunch	roll with poppy seed	water
	S2		
	dinner	yoghurt (mango, passion fruit)	water
	S3	vanilla ice cream with red fruit jelly	water
DAY 2	break-f ast	-	coffee
	S1		
	lunch	Wrap with olives and feta cheese (no garlic!)	fruit-buttermilk, water
	S2		
	dinner	buffalo mozzarella with tomato and olive oil<br>baguette with olives	white wine, water<br>tea
	S3		
SAMPLING DAY	break-f ast	yoghurt	coffee, water
	S1		
	lunch	champignon, parmesan cheese, olive oil, ham, baguette with butter	water
	S2	Cornetto lemon (ice cream)	Fanta
	dinner	champignon, parmesan cheese, olive oil, mozzarella, tomato, ham, baguette with butter;<br>yoghurt	water
	S3		

S1, S2, S3: Snacks.

Table S10. Dietary record—*test person h.*

		Food	Beverages
DAY 1	break-fast	-	water
	S1		
	lunch	bread (with butter)	water
	S2		
	dinner	salad (tomato, cucumber, pepper, ewe's cheese, olives), buttered bread	water, alcohol-free wheat beer
	S3	Raffaelo	
DAY 2	break-fast	-	lemon tea (instant)
	S1		
	lunch	tomato/ mozzarella with balsamic vinegar and oil, bread	water
	S2		coffee with milk
	dinner	flasky pastries with tomato, feta, olives and basil	beer
	S3		
SAMPLING DAY	break-fast	bread with cream cheese and cucumber	water
	S1		water
	lunch	flasky pastries with tomato, feta, olives and basil	water
	S2		water
	dinner	salad with cucumber, tomato and mozzarella, bread with cream cheese and cheese	water
	S3		

S1, S2, S3: Snacks.

Table S11. Dietary record—*test person i.*

		Food	Beverages
DAY 1	break-fast	peanut butter, Nutella and bread	coffee
	S1		tea
	lunch	pasta with mushroom sauce, fruits	tea
	S2	fruits	
	dinner	goulash soup, fruits	tea
	S3		tea
DAY 2	break-fast	peanut butter and bread, fruits	coffee
	S1		
	lunch	pasta witch vegetables and tomato sauce	tea, coffee
	S2	fruits	
	dinner	chicken breast and eggs, fruits	tea
	S3		
SAMPLING DAY	break-fast	bread and cheese, fruits	coffee
	S1		
	lunch	bread and cheese, fruits	tea, coffee
	S2		
	dinner	pasta with tomato sauce, fruits	tea
	S3		

S1, S2, S3: Snacks.

Table S12. Dietary record—*test person j.*

		Food	Beverages
DAY 1	break-fast	chocolate crisped rice with milk	coffee
	S1		
	lunch	pasta with cherry sauce	water
	S2	ice cream with chocolate and nuts	coffee
	dinner	cucumber, pepper, tomato, cheese, sausage and roll	water
	S3		
DAY 2	break-fast	chocolate croissante	coffee
	S1		
	lunch	2 Leberkäse roll with pickled cucumber, 1 ½ blueberry muffin	water
	S2	1 butter biscuit	
	dinner	pasta with paprika pesto and tomatoes, 1 berry-smoothie	water
	S3		
SAMPLING DAY	break-fast	1 banana	coffee, water with juice
	S1		water with juice
	lunch	pasta with paprika pesto and tomatoes, 1 blueberry muffin	water with juice
	S2	1 piece of cake	water with juice
	dinner	gummi bears, potatoes with pepper cream cheese	
	S3		

S1, S2, S3: Snacks.

Table S13. Dietary record—*test person k.*

		Food	Beverages
DAY 1	break-fast	soy yoghurt, pretzel breadstick with cheese	coffee
	S1		
	lunch	casserole with potatoes, carrots, paprika, mincemeat	Spezi (cola and lemonade mix)
	S2	poppy seed strudel	
	dinner		water with lime juice
	S3		
DAY 2	break-fast	poppy seed strudel	coffee
	S1		
	lunch	casserole with potatoes, carrots, paprika, mincemeat	water
	S2		
	dinner	risotto with paprika, tomato and peas	water
	S3		
SAMPLING DAY	break-fast	soy yoghurt	coffee
	S1		
	lunch	risotto with paprika, tomato and peas	water
	S2	gummi bears	Spezi (cola and lemonade mix)
	dinner	pizza margherita	Punica, water
	S3	salt pretzel	

S1, S2, S3: Snacks.

Table S14. Dietary record—*test person l.*

		Food	Beverages
DAY 1	break-fast	chocolate-vanilla pudding	
	S1	bread roll with bratwurst and mustard	Spezi (cola and lemonade mix)
	lunch	casserole with potatoes, carrots, paprika, mincemeat	Spezi (cola and lemonade mix)
	S2		
	dinner	roll with ham and cheese	apple spritzer
	S3		
DAY 2	break-fast	poppy seed strudel	
	S1		
	lunch	casserole with potatoes, carrots, paprika, mincemeat	apple spritzer
	S2		coffee
	dinner	risotto with paprika, tomato and peas	apple spritzer
	S3		
SAMPLING DAY	break-fast	granary bread with ham	
	S1		
	lunch	risotto with paprika, tomato and peas	Spezi (cola and lemonade mix)
	S2	gummi bears	Spezi (cola and lemonade mix)
	dinner	pizza margherita	Punica
	S3		

S1, S2, S3: Snacks.

Table S15. Dietary record—*test person m.*

		Food	Beverages
DAY 1	break-fast	3 slices rye-wheat bread, butter, Nutella, 2 apricots	1 tall cup of coffee with whole milk, 1 glass of ACE-spritzer
	S1		2 glasses of water
	lunch	polenta with stir-fried vegetables (zucchini, pepper, kohlrabi)	1 glass of water
	S2	carrot, nectarine and banana	2 glasses of water
	dinner	5 slices rye-wheat bread (1x with butter, 2x with liverwurst, 2x with cheese	1 glass of water
	S3		2 glasses of water, 1 glas of currant spritzer
DAY 2	break-fast	3 slices rye-wheat bread, 1x liverwurst, 2x butter and marmalade, ½ apricot, ½ nectarine	1 tall cup of coffee with whole milk, 1 glass of currant spritzer
	S1		1 glass of water, 1 glas of currant spritzer
	lunch	3 slices rye-wheat bread with cheese, carrot	1 glass of water
	S2	nectarine, apricot, Hanuta	2 glasses of water
	dinner	whole-grain pasta with minced meat sauce (beef mince, tomato, pepper, peas), cheese	1 glass of water
	S3	Hanuta, 2 Kinder-Schoko-Bons	1 glass of water, 1 glass of tonic
SAMPLING DAY	break-fast	3 slices rye-wheat bread with Nutella, 1 apricot, ½ nectarine	1 tall cup of coffee with whole milk, 2 glasses of water
	S1		2 glasses of water, 1 glass of apple spritzer
	lunch	whole-grain pasta with minced meat sauce (beef mince, tomato, pepper, peas), cheese	1 glass of water
	S2	Hanuta	1 small cup of coffee with milk, 1 glass of water
	dinner		
	S3		

S1, S2, S3: Snacks.

Table S16. Dietary record—*test person n*.

		Food	Beverages
DAY 1	break-fast	3 slices rye-wheat bread	coffee with soy milk
	S1		
	lunch	noodles with olive-tomato sauce (tomato purée, olives, basil, provençal herbs, olive oil, pizza soft cheese)	
	S2	trail mix, gummi bears	
	dinner	hash browns with apple sauce	
	S3		
DAY 2	break-fast	Alnatura breakfast puree with rice milk	nursing tea (fenugreek, fennel, anise, caraway, lemon verbena)
	S1		
	lunch	eggplants with tomato sauce (according to the restaurant without onions and garlic, but with broth from Maggi)	
	S2		
	dinner	bread with margarine and ham, cucumber	
	S3	dark chocolate	
SAMPLING DAY	break-fast	vegan marble cake	coffee with soy milk, Fanta
	S1		
	lunch	bread bun with butter, ham, tomato, cucumber	water
	S2		water
	dinner		
	S3		

S1, S2, S3: Snacks.

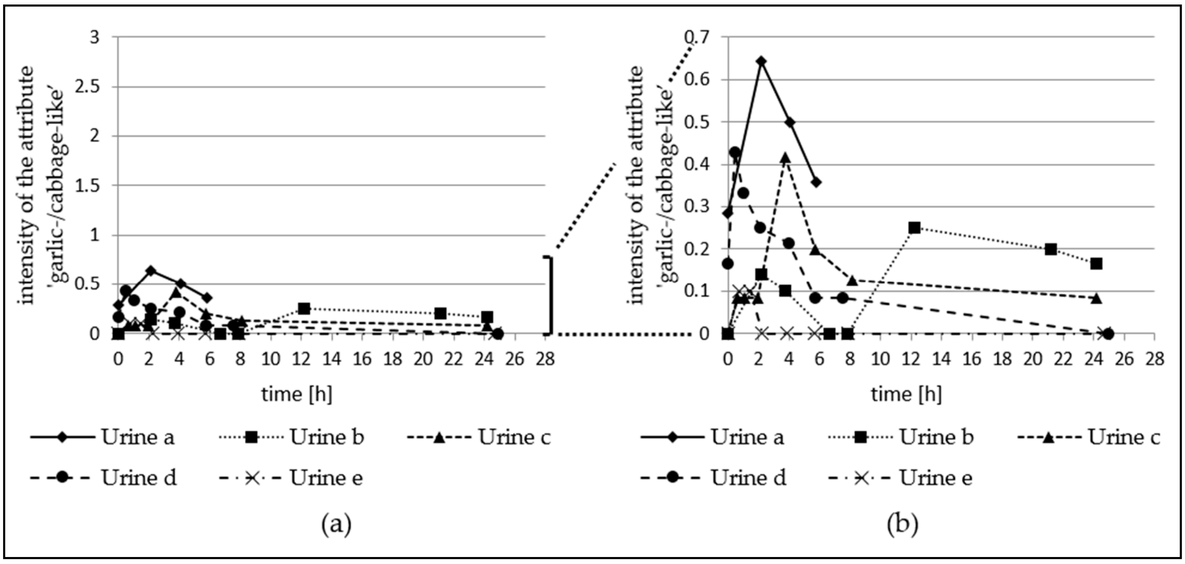

Figure S1. Perceived intensity of a "garlic-/cabbage-like" odor in urine at different intervals before and after ingestion of raw garlic. Up to eight samples were collected before and after ingestion of 30 g (Urine *a*) or 3 g (Urine *b–e)* of raw garlic from different volunteers, whereby sets *a*, *b* and *e* were provided by the same subject but on different days. The first time interval (0 h) corresponds with the urine sampled prior to garlic consumption. Panelists were asked to rate the attribute "garlic-/cabbage-like" on a scale from 0 (no perception) to 3 (strong perception). The values shown represent the mean intensity ratings from all panelists. (**a**): odor intensity on a scale for 0 to 3; (**b**): for better visualization the intensity is presented up to 0.7.

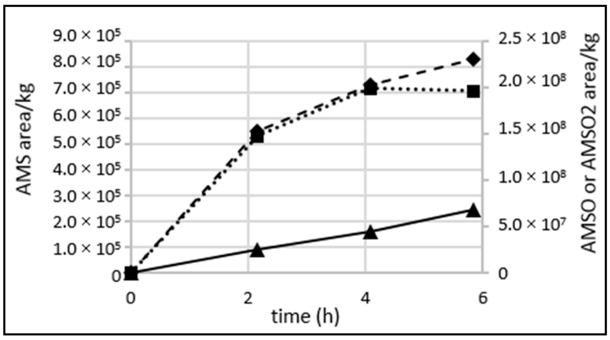

Figure S2. Time-resolved metabolite profiles of AMS, AMSO and $AMSO_2$ for urine set *a*. AMS (◆), AMSO (■), $AMSO_2$ (▲), time 0 h represents the urine sample collected prior to garlic consumption, subsequent time intervals represent urine samples after garlic consumption. Garlic was consumed 4 min after the first urine sample was collected.

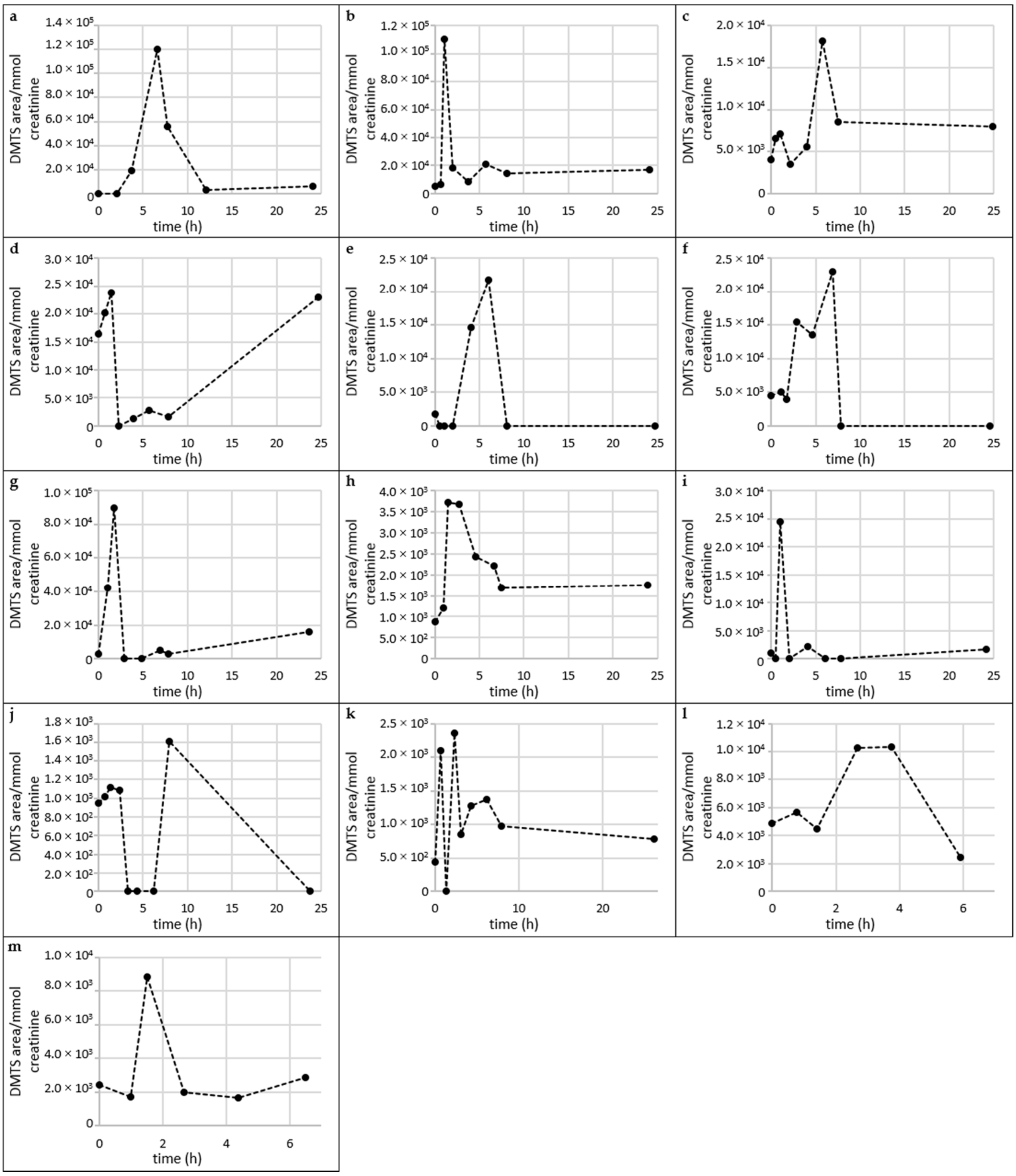

Figure S3. Time-resolved profiles of DMTS-excretion in urine for sets *b* to *n*. Time 0 h represents the urine sample collected prior the garlic consumption, following times represent urine samples after garlic consumption. Garlic was consumed 2 to 38 min after the first urine sample was given. (**a**) set *b;* (**b**) set *c;* (**c**) set *d;* (**d**) set *e;* (**e**) set *f* (**f**) set *g;* (**g**) set *h;* (**h**) set *i;* (**i**) set *j;* (**j**) set *k;* (**k**) set *l;* (**l**) set *m;* (**m**) set *n.*

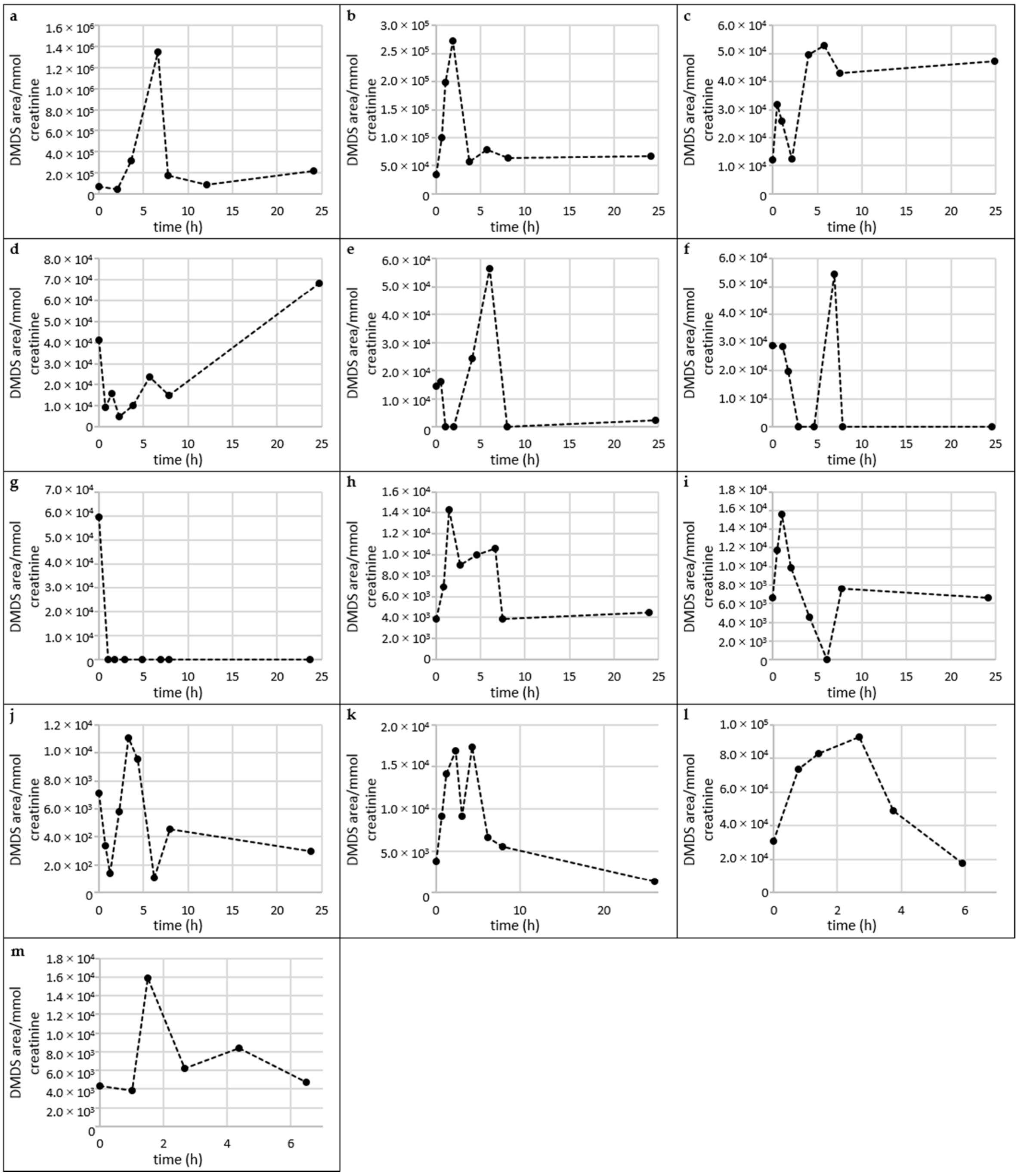

Figure S4. Time-resolved profiles of DMDS-excretion in urine for sets b to n. Time 0 h represents the urine sample collected prior the garlic consumption, following times represent urine samples after garlic consumption. Garlic was consumed 2 to 38 min after the first urine sample was given. (**a**) set *b;* (**b**) set *c;* (**c**) set *d;* (**d**) set *e;* (**e**) set *f* (**f**) set *g;* (**g**) set *h;* (**h**) set *i;* (**i**) set *j;* (**j**) set *k;* (**k**) set *l;* (**l**) set *m;* (**m**) set *n.*

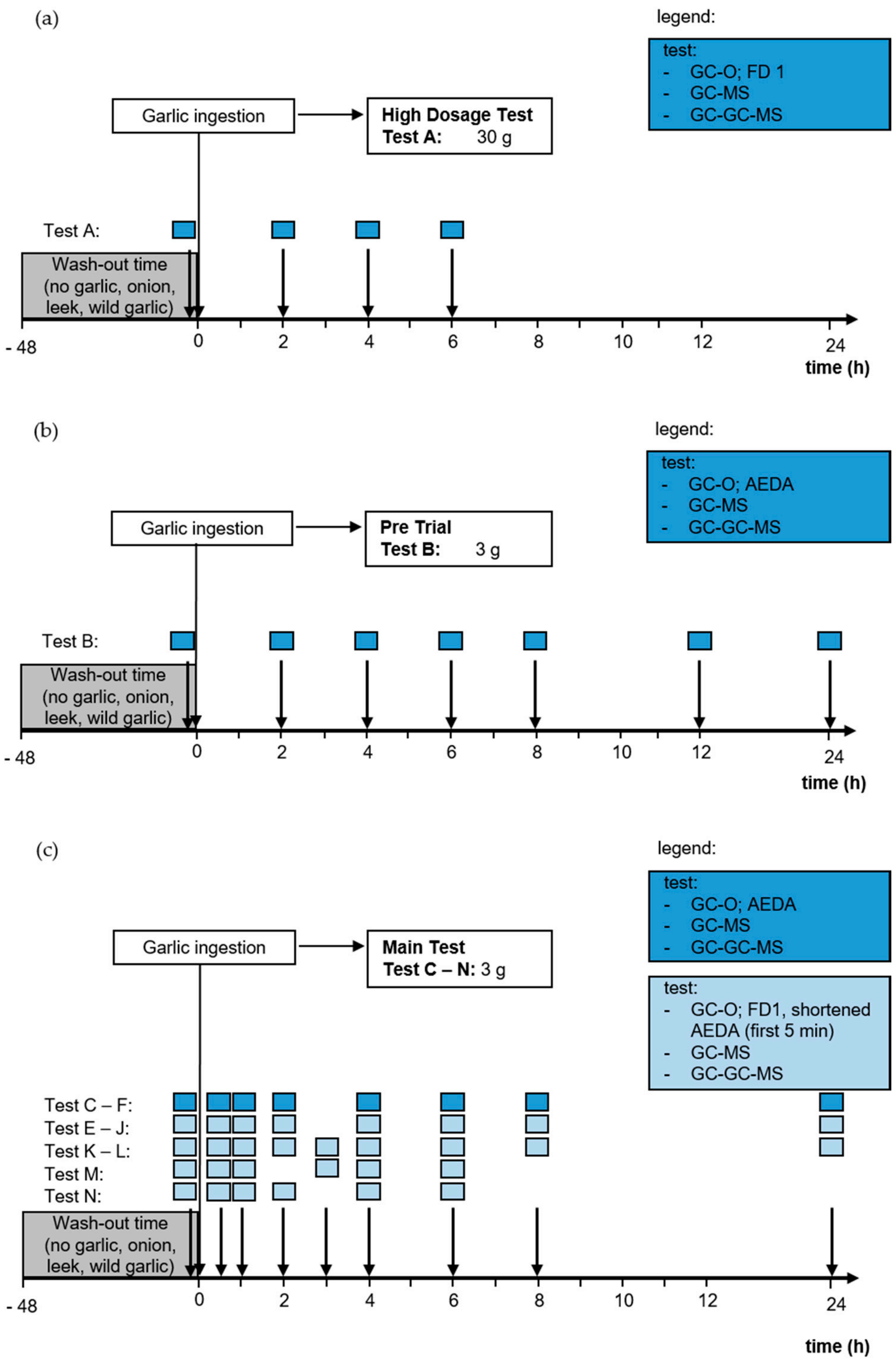

Figure S5. Scheme of urine sampling and analytical methods applied for analysis of the respective samples (**a**) for the high dosage test, (**b**) for the trial test (**c**) for the main test.
